# Neurostimulation improves reading and alters communication within reading networks in dyslexia

**DOI:** 10.1111/nyas.15291

**Published:** 2025-02-01

**Authors:** Sabrina Turker, Philipp Kuhnke, Vincent K. M. Cheung, Konstantin Weise, Gesa Hartwigsen

**Affiliations:** ^1^ Research Group Cognition and Plasticity Max Planck Institute for Human Cognitive and Brain Sciences Leipzig Germany; ^2^ Wilhelm Wundt Institute for Psychology Leipzig University Leipzig Germany; ^3^ Sony Computer Science Laboratories Tokyo Japan; ^4^ Methods and Development Group Brain Networks Max Planck Institute for Human Cognitive and Brain Sciences Leipzig Germany

**Keywords:** dyslexia, effective connectivity, functional magnetic resonance imaging, reading, transcranial magnetic stimulation

## Abstract

The left temporo‐parietal cortex (TPC) is critical for phonological decoding during reading and appears hypoactive in dyslexia. Therefore, a promising approach to alleviating phonological deficits in dyslexia is to modulate left TPC functioning. However, it is unclear how neurostimulation alters activity and network interactions in dyslexia. To address this gap, we combined facilitatory transcranial magnetic stimulation (TMS) to the left TPC in adults with dyslexia with an overt word and pseudoword reading task during functional neuroimaging. We found TMS‐induced improvements in pseudoword reading, reduced contributions of right‐hemispheric regions during reading, and substantial changes between the core reading nodes and an extended network involving the right cerebellum. Stronger coupling between temporo‐occipital and frontal cortices was further directly linked to improvements in pseudoword reading. Collectively, we provide evidence for a crucial role of the left TPC for phonological decoding and show that TMS can successfully modulate reading networks to improve reading in dyslexia.

## Graphical abstract

The left temporo‐parietal cortex is critical for phonological decoding during reading and appears hypoactive in dyslexia. Here, we combined facilitatory neurostimulation to this brain region with functional neuroimaging during word and pseudoword reading in adults with dyslexia. Neurostimulation led to faster pseudoword reading, reduced contributions of right‐hemispheric regions, and substantial changes in effective connectivity between reading nodes and an extended network involving the right cerebellum.

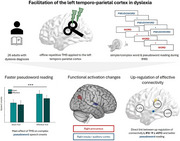

## INTRODUCTION

Literacy plays a foundational role in human communication and learning^1^ and significantly influences personal well‐being and mental health.^2^ However, up to two children in a typical primary school classroom in Germany struggle with literacy acquisition despite adequate schooling and normal intelligence^3^: they have dyslexia, a learning disability affecting reading and writing (International Classification of Diseases, 11th Revision: 6A03.0^4^). Dyslexia leads to severe mental health problems (e.g., depression and anxiety) in every second affected child,^5^, ^6^ causing lower academic achievement and higher unemployment rates.^7^ Although the underlying cause of dyslexia remains unknown, there are various single‐deficit theories^8^ that partially account for the observed differences in phonological, sensorimotor, and visuo‐spatial processing and processing speed.^9^, ^10^ It is largely agreed upon, however, that most individuals with dyslexia have multiple cognitive deficits at the core of their learning disability^11^ that serve as probabilistic predictors.^12^

From a cognitive perspective, dyslexia is characterized by problems in reading and spelling. Individuals most frequently have problems in phonological decoding (i.e., learning and retaining sound–symbol relationships) and accessing (phonological) representations of words.^8^, ^13^ From a neuroscientific perspective, crucial reading areas are hypoactive in children, teenagers, and adults with dyslexia.^14^, ^15^, ^16^, ^17^ One of the most consistent characteristics of dyslexia is hypoactivation of the left temporo‐parietal cortex (TPC),^14^, ^15^, ^16^, ^17^, ^18^ often referred to as the phonological decoding center in the human brain.^19^ Associations between sounds and symbols are formed within this region, which is critical for reading novel words or pseudowords. The left TPC shows heightened neural plasticity during early literacy acquisition^20^ and its activation correlates with individual differences in adult reading beyond reading deficits.^21^ Moreover, its engagement during phonological decoding in struggling readers predicts their response to intervention.^22^, ^23^ Apart from differences in functional activation, neuroimaging studies also provide the first evidence for a strong link between phonological deficits and impaired functional connectivity between the three core reading areas—the left TPC, the left inferior frontal gyrus (IFG), and the left ventral occipito‐temporal cortex (vOTC)—in dyslexia at rest.^24^, ^25^ However, altered network interactions during an explicit reading task have only been investigated in a single study to date.^26^ This study found severe disruptions of functional connectivity in cortico‐cerebellar circuits and between the left TPC and the left vOTC.

Despite decades of research on the concrete symptoms and neural differences that characterize dyslexia, little is known about how this knowledge can help improve treatments for affected individuals. Interventions alleviate reading difficulties by changing underlying neural mechanisms,^27^ but neural changes are highly variable,^28^ and behavioral interventions are only partially successful^29^ and depend upon dosage and outcome measure.^30^ Recent reviews highlight that neuromodulatory interventions, such as transcranial electric stimulation^31^, ^32^, ^33^ and transcranial magnetic stimulation (TMS), constitute promising techniques to successfully modulate reading in typical readers and individuals with dyslexia.^27^, ^34^ Previous studies using transcranial direct current stimulation (tDCS) provide the first evidence for improvements in low‐frequency word, pseudoword, and text reading in dyslexia after facilitation of the left TPC.^32^, ^35^, ^36^, ^37^ To date, there is only one single‐session TMS study in adults with dyslexia where different regions within the left TPC area were targeted. The authors reported improvements in pseudoword reading after the left and right TPC stimulation, and improved word reading after stimulation of the left auditory regions (superior temporal gyrus).^38^ No neurostimulation study in individuals with dyslexia has combined neuroimaging and stimulation, however. There is one study with typical readers in which the authors combined stimulation of the left TPC with neuroimaging and reported that inhibition of the left TPC did not affect reading performance^39^ but increased functional coupling between the left vOTC and the left TPC and altered activation patterns in the left IFG.

In the present study, we applied effective and sham TMS to the left TPC in adults with dyslexia before they read simple and complex words and pseudowords during functional magnetic resonance imaging (fMRI). This allowed us to investigate the immediate after‐effects of neurostimulation at the network level and probe short‐term reorganization in the reading network. Our goal was to improve phonological decoding with potentially facilitatory TMS (intermittent theta burst stimulation), explore TMS‐induced changes in functional activation, and map stimulation‐induced changes at the network level. We hypothesized that facilitation of the left TPC would decrease reading and increase accuracy for pseudowords, with the strongest effect on complex pseudowords because they have the highest phonological decoding demands and are thus more challenging to read, even for compensated adults with dyslexia. Any easier task might be too simple for adults with dyslexia, many of whom received reading training and have developed compensation strategies over decades. Moreover, behavioral improvements would coincide with heightened activity in the core reading areas, resembling activation in typical readers. Finally, we expected that stimulation would alter functional coupling within the reading network due to an up‐regulation of the left TPC that should likely alter, that is, facilitate, communication between reading nodes.

## MATERIALS AND METHODS

### Participants

Participants were young, healthy, right‐handed adults with dyslexia (*N* = 26; 17 females; range: 18–39 years old, *M*_age_ = 26.5) with no prior history of psychiatric, neurological, or hearing disorders. All subjects were German native speakers (no bilingual upbringing) and had either (a) received an official dyslexia diagnosis (reading and spelling deficits) in primary school, or (b) a history of reading and spelling problems and scored at least 1.5 standard deviations below the mean compared to a control group in >50% of all administered reading and spelling tests when compared to a control group (see comparison of performance^26^). All participants had nonverbal intelligence scores within the normal range or above (nonverbal IQ: ≥91^40^) and no prior history of attention deficits, as confirmed by a test assessing continuous attention.^41^

Participants were recruited via flyers and posters distributed in the Max Planck Institute for Human and Cognitive Brain Sciences itself, the university clinic, different buildings of Leipzig University, speech‐language therapists and clinics, and private practices for educational therapy for dyslexia. In addition, the database of the institute was regularly screened for new study subjects with a dyslexia diagnosis. Participants then contacted us via email or telephone and were screened for exclusion criteria for TMS and fMRI, and about their dyslexia diagnosis or their past difficulties in reading and writing. Prior to participation, written informed consent was obtained from each subject. The study was performed according to the guidelines of the Declaration of Helsinki and approved by the local ethics committee at Leipzig University.

### Experimental procedure and behavioral reading assessment

**FIGURE 1:**
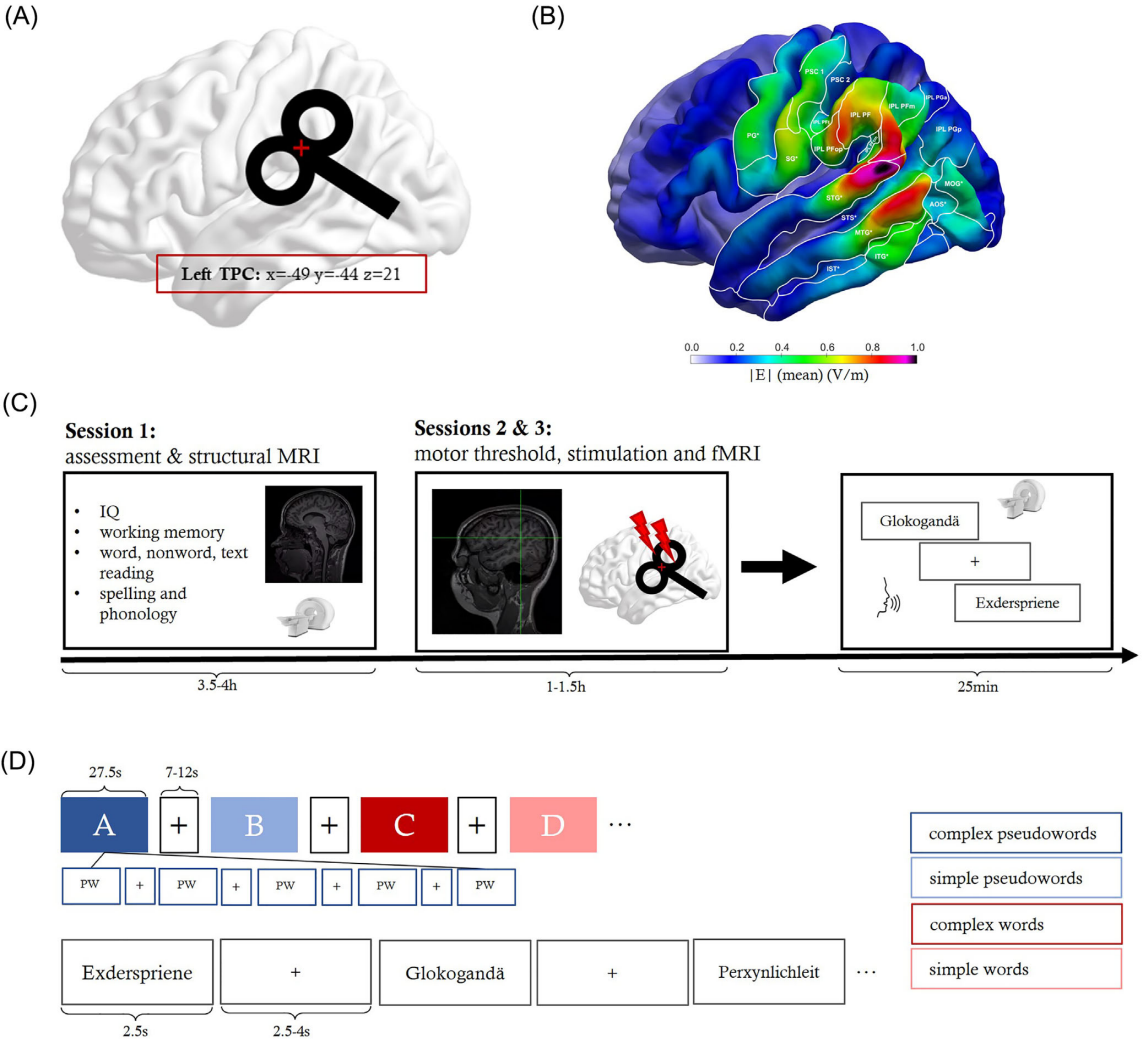
Experimental design and set‐up. (A) The TMS target in the left TPC, as provided in MNI coordinates. (B) The mean electrical field E (in V/m) across participants, showing which portions of the TPC were affected by stimulation. Individual e‐fields can be found in Figure S1. (C) Experiment timeline revealing details on the behavioral assessment and structural MRI in Session 1, as well as motor threshold determination, sham and effective stimulations, and subsequent fMRI in Sessions 2 and 3. (D) Subjects had to overtly read simple and complex pseudowords and words presented for 2.5 s. The inter‐stimulus interval was jittered between 2.5 and 4 s and stimuli were presented in mini‐blocks of five stimuli (27.5 s), with rest periods of 7–12 s between mini‐blocks. Abbreviations: (f)MRI, (functional) magnetic resonance imaging; TMS, transcranial magnetic stimulation; TPC, temporo‐parietal cortex.

The study comprised a 3‐h behavioral testing session and two combined TMS‐fMRI sessions (effective and sham stimulations). During the behavioral testing, we assessed nonverbal intelligence, working memory, reading, and spelling. In the present study, we used common tests for dyslexia diagnosis in childhood to confirm persistent reading and spelling abilities. These comprised standardized childhood dyslexia tests for word and pseudoword reading (SRLT‐II^42^) and rapid automatized naming (TEPHOBE^43^). Additionally, a standardized text comprehension/reading task for schoolchildren in classes 5–12 (LGVT 5–12+^44^), which is the oldest age range in the German school system, was used. The chosen spelling test was developed for teenagers and adults between 15 and 30 years old (Rechtschreibtest^45^). However, since spelling performance does not show an age‐related decline between 30 and 40 years, we administered this test to all participants between 18 and 40 years of age. The two TMS‐fMRI sessions were separated by at least 7 days to prevent carry‐over effects of TMS, and session order (sham or effective) was largely counterbalanced across participants. The study employed a 2×2×2 within‐subject design with the factors TMS (effective stimulation, sham stimulation), stimulus type (words, pseudowords), and complexity (simple stimuli consisting of 2 syllables, complex stimuli consisting of 3–4 syllables). For details of the experimental procedure, stimulation site, and fMRI design, see Figure 1.

### Reading and spelling assessment

We assessed word, pseudoword, and text reading. For word and nonword reading, subjects had to read as many visually presented words and pseudowords as possible within a minute.^42^ Text reading included three scores for fluency/speed (number of words read in 6 min), accuracy (ratio of filled gaps and correct items), and comprehension (number of correctly inserted words).^44^ Norms were available for all three scores.

During our spelling assessment, subjects had to fill in 68 missing words of a text read aloud by the examiner.^45^ Phonemic awareness, lexical access, and retrieval of phonological representations were tested by applying and recording a spoonerism task (German adaption).^46^ Here, subjects had to interchange the initial sounds of verbally presented first names and surnames of 12 well‐known German personalities and cartoon figures (e.g., subjects heard “**D**irk **N**owitzki” and had to say “**N**irk **D**owitzki” as quickly as possible). Response times (times between speech onset and offset) were measured from recordings. As an additional measure for lexical access, we administered a rapid automatized naming task for letters.^43^ A raw score for items read per second was calculated. Characteristics of the included subjects are presented in Table S1. Please note that a behavioral comparison with an age‐matched control group and more details on the specific tests have been published as a separate study.^26^

### Transcranial magnetic stimulation

TMS was applied via a MagPro X100 stimulator (MagVenture). Participants underwent one effective and one sham session. The sham condition mirrored the effective condition in terms of basic set‐up and procedure, but a placebo coil (MCF‐P‐B65) was used, which features the same mechanical outline and acoustic noise as the effective coil but reduces the magnetic field strength by ∼80%. To investigate the causal role of the left TPC for phonological processing and to facilitate reading performance, we applied offline intermittent theta burst stimulation (iTBS), a form of repetitive TMS. It consists of bursts of three pulses at 50 Hz given every 200 ms in a 2‐s stimulation train, repeated every 10 s over 190 s for a total of 600 pulses (for details, see Ref. 47). This protocol has been demonstrated to increase cortico‐spinal excitability^47^ and improve task performance.^48^ Offline protocols can induce adaptive changes in brain activity and connectivity that outlast the stimulation for up to 50 minutes.^49^ The intensity of the stimulation was set to 90% of the individual resting motor threshold. The protocol for assessing the resting motor threshold was conducted in accordance with a standardized procedure.^50^

The MNI coordinates for the left TPC (x/y/z: −49/−44/21) were calculated from three meta‐analyses on hypoactivation in reading areas in dyslexia^14^, ^16^, ^18^ (mean peak of coordinates; see Figure 1). To precisely target these coordinates in each individual participant, they were transformed from MNI to the subject space using SPM12.^51^ We then used stereotactic neuronavigation (TMS Navigator, Localite GmbH) to navigate the coil over the target area and maintain its location throughout stimulation. For neuronavigation, participants’ heads were coregistered onto their T1‐weighted magnetic resonance image before the stimulation sessions. T1 scans were obtained beforehand with a 3T MRI scanner (Siemens) using an MPRAGE sequence (176 slices in sagittal orientation; repetition time: 2.3 s; echo time: 2.98 ms; field of view: 256 mm; voxel size: 1×1×1 mm; no slice gap; flip angle: 9°; phase encoding direction: A/P). The procedure was as follows: after neuronavigation to the target position, subjects received the 180s iTBS protocol before directly being taken to the MRI in a wheelchair with as little communication as possible. We made sure that, for all subjects, the time between the end of the stimulation and the onset of the task was below 6 min 30 seconds.

### Functional neuroimaging

We used an event‐related mini‐block design containing 400 words and pseudowords. In each session, participants overtly read a random compilation of 100 words (50 simple, 50 complex) and 100 pseudowords (50 simple, 50 complex) in blocks of 5 stimuli per mini‐block (see Figure 1D). No stimulus was repeated across sessions. The simple word stimuli consisted of two syllables and 4–6 letters and were taken from Schuster et al.^52^ For complex words, we chose the 100 most frequent 3‐ and 4‐syllabic words (10–14 letters) from the dlex database (http://www.dlexdb.de/). Pseudowords were then created using *Wuggy* (http://crr.ugent.be/programs‐data/wuggy) based on the simple and complex word lists. We included orthographic neighborhood and lexical frequency for all items as covariates in behavioral analyses. Stimuli were presented in white font (Arial, font size 40) on a black screen.

During fMRI, stimuli were presented for 2.5 seconds. We jittered the between‐stimulus interval (2.5–4 s), as well as the between‐mini‐block interval (7–12 s; see Figure 1). Subjects were instructed to read out all stimuli as fast and correctly as they could with as little head movement as possible. Subjects’ in‐scanner responses were recorded and manually analyzed using *Audacity* 3.0.0^53^ and *Praat* 6.1.38^54^ by four independent raters, two analyzing each audio file in 50% of cases. We computed an interrater reliability of >0.85. The accuracy for all trials was checked by a third person. Speech onsets comprised the time from stimulus presentation to the start of overt reading, and overt reading times comprised the full duration until the stimulus was read aloud.

Functional MRI data were collected on a 3T Siemens Magnetom Skyra scanner (Siemens) with a 32‐channel head coil. Blood oxygenation level‐dependent (BOLD) images were acquired with a gradient‐echo BOLD Echo Planar Imaging (EPI) sequence (repetition time [TR]: 2 s, echo time [TE]: 22 ms; flip angle: 80°; field of view [FoV]: 204 mm; voxel size: 2.5 × 2.5 × 2.5 mm; bandwidth: 1794 Hz/Px; phase encoding direction: A/P; multiband acceleration factor: 3). B0 field maps were acquired for susceptibility distortion correction using a spin‐echo EPI sequence (TR: 8000 ms; TE: 50 ms; flip angle: 90°; bandwidth: 1794 Hz/Px).

### Data analyses

#### Data availability

All data is available via the OSF registry https://osf.io/sgpwj.

#### Behavioral analyses

Statistical analyses were performed with *JASP* 0.17.3.0^55^ and *R* 4.1.3^56^. Speech onsets, overt reading time, and response accuracy were analyzed with generalized linear mixed models (GLMMs) using *glmmTMB* 1.1.3 in *R* 4.1.3^56^ as done in a previous TMS study using the exact same task and set‐up.^39^ Speech onsets and reading times did not satisfy normality assumptions; both distributions appeared to be positively skewed. To avoid data transformation, we fitted the models of speech onset and reading time via gamma distributions with the identity link function. Response accuracy was modeled as a binary response, meaning that each trial could either be correct or incorrect. Thus, the GLMM was fitted with the binomial distribution and logit link function. All models included as fixed effects the four‐way interaction of TMS (effective vs. sham), stimulus type (word vs. pseudoword), complexity (simple vs. complex), and session order (sham vs. effective in the first session), and all lower order terms since all of them were variables of interest. Additionally, we included the orthographical neighborhood (OLD20; a measure of orthographic similarity providing information on how distant one word is from its closest neighbors^57^) and lexical frequency as fixed effects to control for potential effects due to differences in these two linguistic parameters. Regarding the random effects, we used a maximal random effect structure with the subject as the grouping variable to avoid inflated Type I errors and performed model selection by moving from maximal to minimal models, selecting the maximal model that converged. Model comparison was performed using the Akaike information criterion. The significance of the variables was evaluated using the Type III Wald test. Marginal effects were calculated using a step‐down simple effects analysis. Regarding the random effects, we used a crossed maximal random effect structure with the subject and word‐item as grouping variables. Our models thus included random slopes for TMS, stimulus type, complexity, and session order, as well as a random intercept for word‐item. The significance of the variables was evaluated using Wald's test and marginal effects were calculated using the emmeans package.

To analyze stimulus‐specific recruitment of the left TPC regions, multiple repeated‐measure ANOVAs were computed with *JASP*.^55^ These 2×2×2 ANOVAs included TMS (sham vs. effective), stimulus type (word vs. pseudoword), and complexity (simple vs. complex) as fixed factors and brain activation in the left TPC subregions (beta values) as dependent variables. For significant interactions, post‐hoc tests were computed and Holm–Bonferroni correction was applied in *JASP*.

Earlier TMS studies that aimed to modulate reading performance had reported strong TMS effects with Cohen's *d* ranging from −0.37 to −1.96 based on repeated‐measure ANOVAs with 10 subjects.^38^, ^58^ Similarly, work from our own group revealed strong TMS effects after the left TPC stimulation in a group of 26 subjects (Cohen's *d* = 0.63/*η* ^2^ = 0.09).^59^ Since no power analysis had been performed before the onset of the study, we used G‐power to perform a post‐hoc sensitivity calculation, which reveals the smallest effect that could have been reliably detected with a high probability given our sample size (*N* = 26). The post‐hoc sensitivity analysis showed that assuming *α* = 0.05, we had 80% power to detect effect sizes larger than *d* = 0.57 (*η* ^2^ = 0.075) for two‐tailed *t*‐tests and larger than *d* = 0.47 (*η* ^2^ = 0.052) for repeated‐measure ANOVAs.^60^

#### Preprocessing, univariate, and multivariate functional activation analyses

Preprocessing was performed using *fMRIprep* (20.2.1).^61^ Anatomical T1‐weighted images were corrected for intensity nonuniformity (using *N4BiasFieldCorrection* from ANTs 2.3.3,^62^ skull‐stripped (using *antsBrainExtraction* from ANTs 2.3.3), segmented into gray matter, white matter, and cerebrospinal fluid (using *fast* in FSL 5.0.9),^63^ and normalized to MNI space (MNI152NLin2009cAsym; using *antsRegistration* in ANTs 2.3.3). Brain surfaces were reconstructed using *reconall* (FreeSurfer 6.0.1).^64^

Functional BOLD images were coregistered to the anatomical image (using *bbregister* in FreeSurfer 6.0.1), distortion corrected based on B0‐field maps (using *3dQwarp* in AFNI 20160207),^65^ slice‐timing corrected (using *3dTshift* from AFNI 20160207), motion corrected (using *mcflirt* from FSL 5.0.9), normalized to MNI space (via the anatomical‐to‐MNI transformation), and smoothed with a 5 mm^3^ FWHM Gaussian kernel (using *SPM12*; Wellcome Trust Centre for Neuroimaging; http://www.fil.ion.ucl.ac.uk/spm/).^51^ Moreover, physiological noise regressors were extracted using the anatomical version of *CompCor* (aCompCor).^66^

We performed a whole‐brain random‐effects group analysis based on the general linear model (GLM), using the two‐level approach in *SPM12*. First, individual participant data were modeled separately. The participant‐level GLM included regressors for the four experimental conditions (simple words, complex words, simple pseudowords, and complex pseudowords), modeling trials as box car functions (2.5 s duration) convolved with the canonical hemodynamic response function. Only correct trials with given responses were analyzed; incorrect trials were modeled in a separate regressor‐of‐no‐interest. To control for movement artifacts, we included 24 motion regressors (the 6 base motion parameters + 6 temporal derivatives of the motion parameters + 12 quadratic terms of the motion parameters and their temporal derivatives).^67^ Moreover, we performed motion scrubbing to remove individual time points with a strong volume‐to‐volume movement from the analysis.^68^ To this end, we computed framewise displacement as a measure of excessive volume‐to‐volume movement and added individual regressors for volumes that exceeded a threshold of >0.9, as proposed for task‐based fMRI data.^69^ Finally, we included the top 10 aCompCor regressors explaining the most variance in physiological noise.^66^ The data were subjected to an AR(1) auto‐correlation model to account for temporal auto‐correlations, and high‐pass filtered (cutoff 128 s) to remove low‐frequency noise.

Contrast images for each participant were computed at the first level. At the second (group) level, these contrast images were submitted to one‐sample or paired *t*‐tests (to test for interactions). For all second‐level analyses, a gray matter mask was applied, restricting statistical tests to voxels with a gray matter probability >0.1 (MNI152NLin2009cAsym gray matter template in *fMRIprep*). All activation maps were thresholded at a voxel‐wise *p* < 0.001 and a cluster‐wise *p* < 0.05 FWE‐corrected.

To test for stimulus‐specific recruitment in the left TPC region, we investigated the activation magnitude through the extraction of beta values in five designated regions of interest (ROIs) that had been differentially engaged in the word versus pseudoword contrasts. We included the left angular gyrus (AG), the left pSTG, the left posterior supramarginal gyrus (SMG; henceforth pSMG), the left anterior SMG (aSMG), and the left SPL from the Harvard‐Oxford atlas in FSL.^70^, ^71^, ^72^, ^73^ ROIs were thresholded at >30% probability of belonging to the respective anatomical region and binarized.

A major shortcoming of univariate analyses is the low sensitivity on the individual subject level.^74^ Specifically, they are sensitive to absolute changes in the mean activation magnitude of brain regions, while multivariate pattern analyses (MVPAs) are sensitive to relative activation differences between voxels.^75^ Therefore, we complemented our standard univariate analyses with an MVPA using *The Decoding Toolbox* ^76^ implemented in MATLAB (version 2021a). We performed searchlight MVPA, moving a spherical region of interest of 5 mm radius through the entire brain.^77^ At each searchlight location, a machine‐learning classifier (an L2‐norm support vector machine; C = 1) aimed to decode between effective and sham stimulation. We performed leave‐one‐participant‐out cross‐validation (CV), training on the activation patterns from *n*−1 participants and testing on the left‐out participant. Activity patterns comprised beta estimates for each mini‐block of every participant. For statistical inference, we performed a permutation test across the accuracy‐minus‐chance maps of the different CV‐folds (using *SnPM13* ^78^), thresholded at a voxel‐wise *p* < 0.001 and a cluster‐wise *p* < 0.05 FWE‐corrected.

#### Dynamic causal modeling

We performed dynamic causal modeling (DCM) to assess directed causal influences between reading‐related brain regions.^79^ DCM estimates a model of effective connectivity between brain regions to predict a neuroimaging time series^80^ and consists of three types of parameters: (1) intrinsic directed connections between brain regions (i.e., connections that are not influenced by the specific task); (2) modulatory inputs that change connection strengths during a certain experimental manipulation (e.g., words and pseudowords may influence intrinsic connections differently); and (3) driving inputs that engage individual network nodes (here, all written stimuli). The goal of DCM is to optimize a tradeoff between model fit (of the predicted to observed time series) and complexity (deviation of model parameters from their prior expectations), measured by the model evidence.^81^

We performed a two‐level analysis using SPM12 implemented in MATLAB.^82^ At the first level, a full model was specified and estimated for each participant and TMS condition (effective, sham). The first full model included the classical reading network from literature: the left TPC, the left vOTC, and the left IFG. These regions were defined functionally in each individual participant as the top 10% most active voxels for [all trials > rest] within 20 mm spheres around the MNI peak coordinates from the meta‐analysis by Martin et al.^83^: the left TPC = −49 −44 21; the left IFG = −52 20 18; the left vOTC = −42 −68 −22. The second full model included the extended reading network based on hypoactive brain areas as revealed in earlier analyses of the sham data.^26^ The respective areas were the left SMG, the left vOTC, the right vOTC, and the right cerebellum. Again, these regions were defined functionally in each individual participant as the top 10% of most active voxels for [all trials > rest] within the hypoactive group clusters. The full models assumed that all ROIs were fully connected via reciprocal connections. The first eigenvariate of the BOLD time series of each region was extracted and adjusted for effects of interest (all experimental conditions) using our participant‐level GLM. DCM inputs were mean‐centered, so that the intrinsic connections reflected the mean connectivity across experimental conditions.^81^

At the second level, we performed three group‐level analyses using parametric empirical Bayes and Bayesian model reduction (BMR), modeling effective connectivity during (1) sham TMS, (2) effective TMS, and (3) the contrast between effective versus sham TMS. In each group‐level analysis, DCM parameters of individual participants were entered into a GLM that decomposed interindividual variability in connection strengths into group effects and random effects.^84^ BMR then compared the full model against reduced models that had parameters switched off that did not contribute to the model evidence (i.e., prior mean and variance set to 0).^82^ For group‐level inferences, we computed the Bayesian model average (BMA), the average of parameter values across models weighted by each model's posterior probability (Pp).^85^ We chose the BMA approach as the BMA accommodates uncertainty about the true underlying model.^82^ The BMA was thresholded to only retain parameters with a Pp > 95%.^84^

To determine whether the observed connectivity changes were directly linked to the behavioral improvement in pseudoword reading, we computed standard Pearson correlations between significant DCM parameters and behavioral improvements for each DCM separately using *JASP* v0.17.3.0.^55^ To this end, subject‐specific modulations of IFG‐vOTC connectivity during pseudoword reading were correlated with behavioral improvements in complex pseudoword reading performance. The observed greater facilitation of this connection (indicated by positive values) was directly linked to an improvement in complex pseudoword reading speech onsets (as indicated by a negative value). In other words, the stronger the modulation of the connectivity between IFG‐vOTC for pseudowords was, the faster the subjects were at reading complex pseudowords after the left TPC stimulation.

## RESULTS

The main aims of the present study were to explore TMS‐induced changes in reading performance, functional activation, and functional coupling between the classical reading areas. Last, we aimed to understand which neural changes were directly linked to reading performance to help inform future neurostimulation studies.

### TMS‐induced changes in reading performance

To assess the expected positive effect of effective TMS on in‐scanner reading performance, speech onset, overt reading time (speech production), and the accuracy of each trial were modeled using GLMMs. Although our main hypothesis was that TMS should mainly improve the task relying heavily on phonological decoding (i.e., speech onsets for complex pseudoword reading), we were also keen on exploring potential overall effects of TMS and its interactions with session order, complexity of stimuli, and stimulus type across our assessed variables. The results of these first GLMMs are presented in Tables 1, 2, 3 (see post‐hoc comparisons in Tables S2 and S3).

**TABLE 1:** Linear mixed model results displaying the effect of transcranial magnetic stimulation (TMS) and stimulus properties on speech onsets.

**Predictor**	***χ* ^2^**	**Df**	***p*‐value**
Intercept	344.775	1	< 2.2e‐16^***^
TMS	4.170	1	0.054^+^
Complexity	17.214	1	4.11e‐05^***^
Stimulus type	80.192	1	< 2.2e‐16^***^
Session	0.856	1	0.256
OLD20	0.019	1	0.936
Lexical frequency	5.840	1	0.030^*^
TMS: Complexity	0.682	1	0.331
TMS: Stimulus type	0.483	1	0.373
Complexity: Stimulus type	0.057	1	0.179
TMS: Session	3.564	1	0.065^+^
Complexity: Session	0.076	1	0.723
Stimulus type: Session	5.284	1	0.014^*^
TMS: Complexity: Stimulus type	0.184	1	0.506
TMS: Complexity: Session	0.348	1	0.494
TMS: Stimulus type: Session	2.133	1	0.109
Complexity: Stimulus type: Session	2.837	1	0.009^**^
TMS: Complexity: Stimulus type: Session	0.179	1	0.739

*Note*: Speech onsets of each trial were modeled to follow a gamma distribution using a generalized linear mixed model. The significance of predictors was assessed using the Wald test.

*p < 0.05; **p < 0.01; ***p < 0.001, + does not meet the conventional threshold we applied but indicates a potential trend.

**TABLE 2:** Linear mixed model results displaying the effect of transcranial magnetic stimulation (TMS) and stimulus properties on overt reading times (from speech onset to speech offset).

**Predictor**	***χ* ^2^**	**Df**	***p*‐value**
Intercept	602.688	1	< 2.2e‐16^***^
TMS	0.932	1	0.361
Complexity	47.796	1	2.588e‐11^***^
Stimulus type	21.839	1	3.18e‐06^***^
Session	1.002	1	0.428
OLD20	13.134	1	<0.001^***^
Lexical frequency	2.196	1	0.271
TMS: Complexity	2.493	1	0.107
TMS: Stimulus type	4.118	1	0.011^*^
Complexity: Stimulus type	546.808	1	<2.2e‐16^***^
TMS: Session	0.120	1	0.872
Complexity: Session	0.214	1	0.596
Stimulus type: Session	0.465	1	0.503
TMS: Complexity: Stimulus type	7.491	1	<0.001^***^
TMS: Complexity: Session	0.193	1	0.793
TMS: Stimulus type: Session	3.144	1	0.041^*^
Complexity: Stimulus type: Session	24.242	1	8.694e‐09^***^
TMS: Complexity: Stimulus type: Session	15.852	1	1.787e‐06^***^

*Note*: Reading times of each trial were modeled to follow a gamma distribution using a generalized linear mixed model. The significance of predictors was assessed using the Wald test.

*p < 0.05; ***p < 0.001.

**TABLE 3:** Linear mixed model results displaying the effect of transcranial magnetic stimulation (TMS) and stimulus properties on reading accuracy.

**Predictor**	***χ* ^2^**	**Df**	***p*‐value**
Intercept	121.734	1	<2.2e‐16^***^
TMS	1.448	1	0.213
Complexity	0.792	1	0.266
Stimulus type	19.448	1	1.433e‐05^***^
Session	1.872	1	0.211
OLD20	0.174	1	0.681
Lexical frequency	0.020	1	0.808
TMS: Complexity	0.700	1	0.335
TMS: Stimulus type	2.194	1	0.186
Complexity: Pseudoword	10.225	1	0.006^**^
TMS: Session	5.881	1	0.014^*^
Complexity: Session	3.384	1	0.096
Stimulus type: Session	3.259	1	0.092
TMS: Complexity: Stimulus type	1.785	1	0.190
TMS: Complexity: Session	4.814	1	0.025^*^
TMS: Stimulus type: Session	6.350	1	0.014^*^
Complexity: Stimulus type: Session	5.684	1	0.029^*^
TMS: Complexity: Stimulus type: Session	7.437	1	0.007^**^

*Note*: Response accuracies for each trial were modeled using a binomial generalized linear mixed model. The significance predictors were assessed using the Wald test.

*p < 0.05; **p < 0.01; ***p < 0.001.

For speech onsets, we found a statistical trend for the effect of TMS (*χ*2 *=* 3.715, *p* = 0.054) and strong main effects for complexity (*χ*2 *=* 16.819, *p* < 0.001), stimulus type (*χ*2 *=* 87.527, *p* < 0.001), and lexical frequency (*χ*2 *=* 4.721, *p* = 0.03). We found that speech onsets were shorter (i.e., subjects read faster) after effective compared to sham stimulation and more complex stimuli required more time to be read. In terms of stimulus type, words were always read faster and a higher lexical frequency was linked to faster reading. There were no significant interactions with TMS. Our initial hypothesis was that stimulation would improve phonological decoding, which should be most visible in complex pseudowords. Therefore, we also computed a model to assess the potential impact of TMS on complex pseudowords, the condition that showed the strongest differences between the current sample of adults with dyslexia and typical readers in an earlier comparison.^26^ The new GLMM contained only speech onsets of complex pseudowords, and data were modeled with the fixed factors TMS, session order, orthographic neighborhood, and spelling performance. We found main effects of TMS (*χ*2 = 11.038, *p* < 0.001) and of spelling performance (*χ*2 *=* 8.980, *p* = 0.009) on complex pseudoword speech onsets. These analyses confirmed that effective stimulation led to faster speech onsets for complex pseudowords and better spelling performance was linked to faster speech onsets (please note that models with spelling and reading performance in the other LMMs did not converge).

For overt reading times, there were significant main effects of complexity (*χ*2 *=* 44.466, *p* < 0.001), stimulus type (*χ*2 *=* 21.705, *p* < 0.001), and orthographic neighborhood (*χ*2 *=* 12.412, *p* < 0.001), where stimuli with higher OLD20 (orthographic Levenshtein distance 20) required longer overt reading times. Complex items and pseudowords as stimulus types required longer overt reading times. Additionally, the GLMM revealed a significant interaction of TMS by complexity by stimulus type by session (*χ*2 = 22.812, *p* < 0.001). Post‐hoc tests revealed selective differences between effective and sham for complex pseudowords, with performance always being better in the second session (active in the first session: *z* = 6.301, *p* < 0.0001; active in the second session: *z* = −5.317, *p* < 0.0001). This indicates a strong learning effect for overt reading times of complex pseudowords.

**FIGURE 2:**
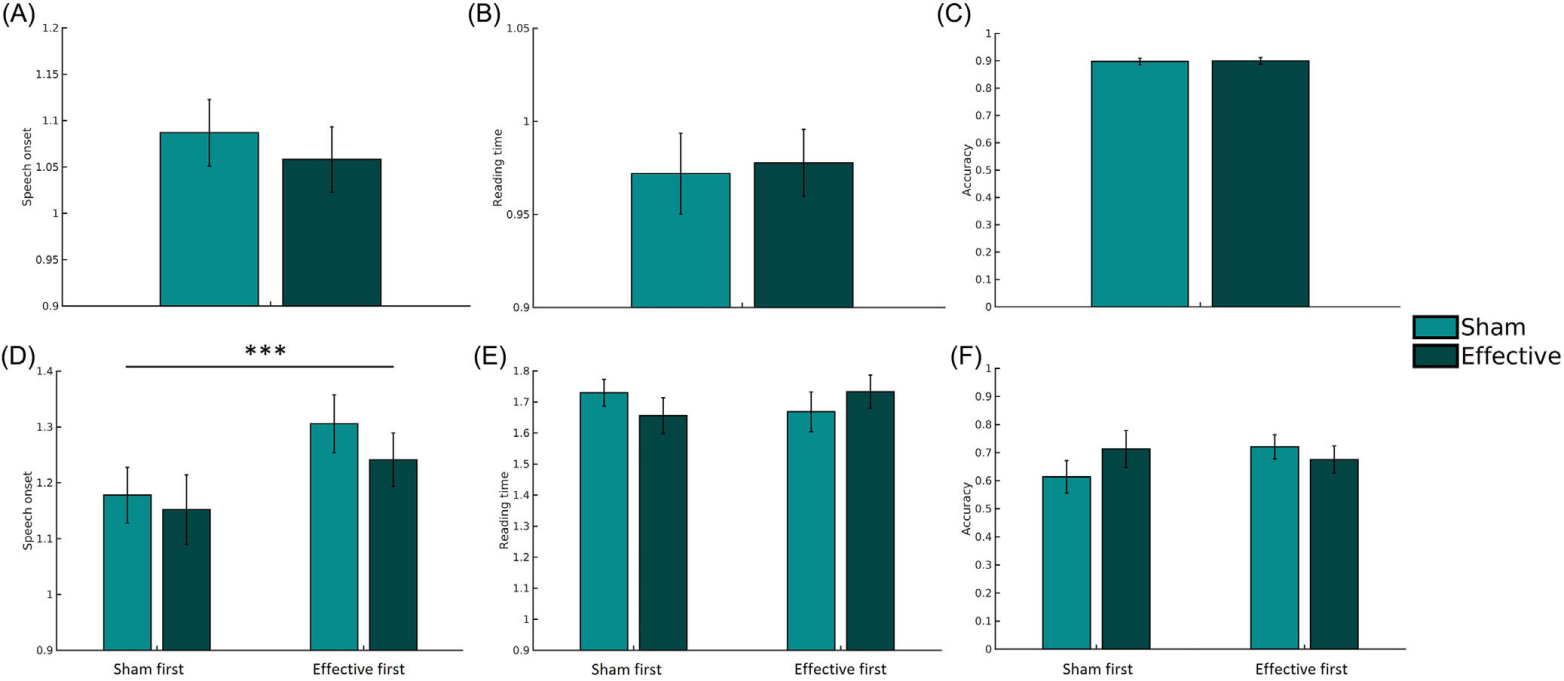
Visualization of behavioral results and GLMMs. Top row, results for all stimuli: (A) speech onsets, (B) overt reading times, and (C) reading accuracy. The weak main effect of TMS on speech onsets across stimuli is indicated. Bottom row, complex pseudowords: (D) speech onsets, (E) reading times, and (F) accuracy. We further show a strong effect of TMS on complex pseudoword speech onsets regardless of the session order, but not for complex pseudoword reading times or accuracy. ^+^ *p* = 0.054 (trend); ^***^ *p* <0.001. Abbreviations: GLMMs, generalized linear mixed models; TMS, transcranial magnetic stimulation.

For reading accuracy, we found a significant main effect of stimulus type (*χ*2 *=* 18.824, *p* < 0.001) and a significant interaction of TMS by complexity by stimulus type by session (*χ*2 *=* 7.242, *p* = 0.007). Overall, subjects with dyslexia had a significantly lower accuracy for pseudowords, and disentangling the significant interaction, we found that accuracy differences were only significant for simple words when stimulation was delivered in the second session (*t ratio* = 2.175, *p* = 0.03) and for complex pseudowords when stimulation was delivered in the second session (*t ratio* = 2.217, *p* = 0.027). This could indicate an effect of TMS on learning (see Discussion). The GLMM results are displayed in Figure 2.

### TMS decreases task‐related activity in right‐hemispheric areas outside the reading network

**FIGURE 3:**
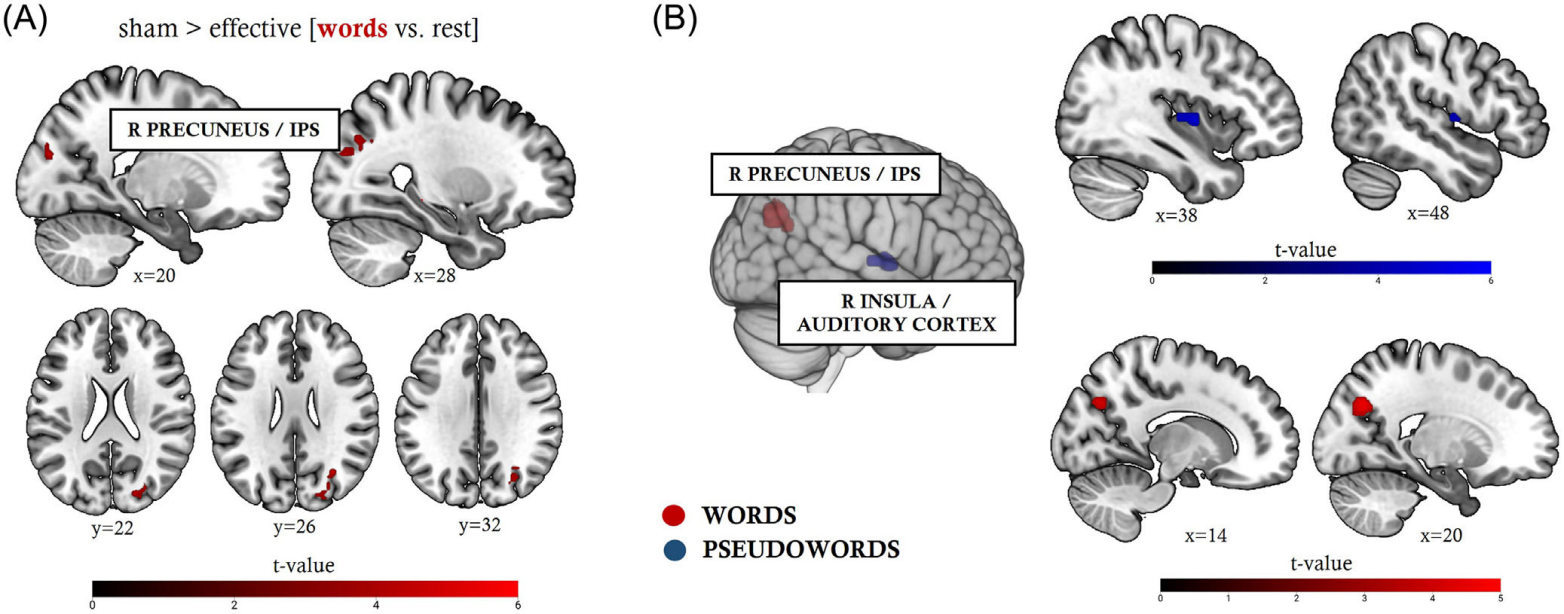
The effects of effective TMS over the left TPC on whole‐brain functional activation. (A) Univariate and (B) multivariate analyses show altered contributions of the right precuneus/right IPS and the right insula/auditory cortex during word and pseudoword reading, respectively (FWE cluster‐level corrected at *p* < 0.05 and voxel‐level corrected at *p* < 0.001). Abbreviations: IPS, intraparietal sulcus; TMS, transcranial magnetic stimulation; TPC, temporo‐parietal cortex.

At the whole brain level, we did not find any significant modulation in the stimulated area after TMS. However, univariate analyses revealed that effective stimulation as compared to sham stimulation resulted in a word‐specific activation decrease in a cluster situated in the right superior lateral occipital cortex (67 voxels; peak: x/y/z = 30/−65/29), covering portions of the right precuneus (Figure 3A). Cytoarchitectonically, this cluster is tied to the intraparietal sulcus (IPS). In other words, after the facilitation of the left TPC, functional activation in this region (often also subsumed as the posterior parietal cortex) decreased when subjects read simple and complex words. This finding was confirmed in multivariate analyses, where activation patterns during word reading in a virtually identical cluster within the right IPS/precuneus enabled above‐chance decoding between effective and sham TMS across subjects (96 voxels; peak: x/y/z = 20/−68/34; *t* = 4.71).

Univariate analyses did not yield any significant differences between effective and sham sessions for pseudoword reading. The more sensitive multivariate analysis, however, revealed a TMS‐induced shift in activation patterns in the right insula/auditory cortex (65 voxels; peak: x/y/z = 43/−8/7; *t* = 5.42) during pseudoword reading (Figure 3B).

### TMS‐induced differences in effective connectivity

**FIGURE 4:**
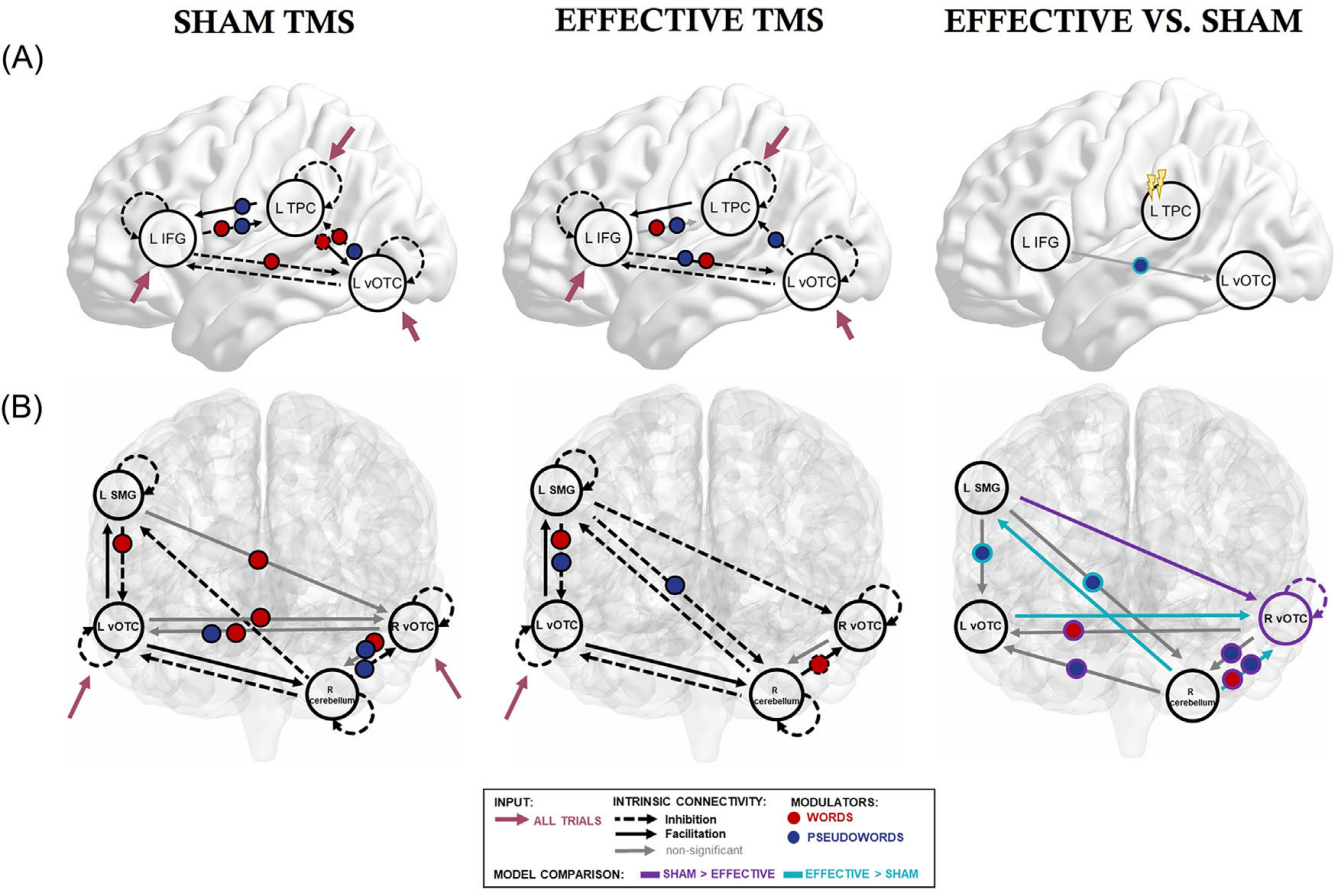
TMS‐induced differences in effective connectivity within (A) the classical reading network and (B) the extended, hypoactive reading network. For each panel, we present the corresponding significant interactions and modulations for sham, effective, and effective versus sham. Only findings with a posterior probability >0.95 are shown. Abbreviations: IFG, inferior frontal gyrus; L, left; R, right; SMG, supramarginal gyrus; TMS, transcranial magnetic stimulation; TPC, temporo‐parietal cortex; vOTC, ventral occipito‐temporal cortex.

Next, we explored whether effective TMS altered communication within the classical reading network and within the extended reading network based on hypoactive brain regions (Figure 4). Detailed results for all six models are provided in Tables S4–S9.

Our first full DCM assessed interactions within the classical reading network: the left IFG, the left TPC, and the left vOTC (Figure 4A). Focusing on the TMS‐induced changes in network interactions within this network, we found that, relative to sham TMS, effective TMS over the left TPC increased the facilitatory modulation of pseudowords on the connection from the left IFG to the left vOTC (sham: estimate = 0.295, Pp = 0.904, Hz change = −0.033; effective: estimate = 1.018, Pp = 1, Hz change = 0.103). This pathway is part of the so‐called ventral (sight word) reading route. To explore whether this change was behaviorally relevant, we computed a Pearson correlation between the change in IFG‐vOTC modulation by pseudowords (effective—sham) and the improvement in speech onsets of complex pseudowords (effective—sham), which was significant (*r* = −0.474, *p* = 0.014, Fisher's *z* = −0.515). In other words, a stronger modulation of functional coupling from the left IFG to the left vOTC by pseudowords was associated with a larger facilitation of speech onsets for complex pseudowords.

While the significant changes in the classical reading network were reduced to a single modulation, effective TMS led to several significant changes within an extended, hypoactive reading network (Figure 4B). Effective TMS led to a stronger modulation by pseudowords of coupling from the left SMG to the left vOTC [sham: estimate = <0.0001, Pp = 0, Hz change = −0.238; effective: estimate = 0.685, Pp = 1, Hz change = 0.363] and from the left SMG to the right cerebellum [sham: estimate = <0.0001, Pp = 0, Hz change = 0; effective: estimate = 0.376, Pp = 1, Hz change = 0.253], suggesting that TMS facilitated communications from the left SMG to these two areas. TMS further lowered pseudoword‐specific modulations of functional coupling from the right cerebellum to the left vOTC [sham: estimate = 0.598, Pp = 1, Hz change = 0.334; effective: estimate = <0.0001, Pp = 0, Hz change = −0.118] and to the right vOTC [sham: estimate = 0.496, Pp = 0.99, Hz change = 0.319; effective: estimate = <0.0001, Pp = 0, Hz change = 0.131]. The latter was also the case for modulations by words [sham: estimate = <0.0001, Pp = 0, Hz change = −0.177; effective: estimate = −0.340, Pp = 1, Hz change = −0.209]. Overall, intrinsic connectivity from the right cerebellum to the right vOTC was altered so that TMS turned this connection into facilitation [sham: estimate = −0.092, Pp = 1; effective: estimate = 0.073, Pp = 1]. During effective TMS, there was no significant interaction between the right vOTC and the right cerebellum for pseudowords [sham: estimate = 0.443, Pp = 1, Hz change = 0.312; effective: estimate = <0.0001, Pp = 0, Hz change = 0], and from the right vOTC to the left vOTC for words [sham: estimate = 0.308, Pp = 0.953, Hz change = 0.083; effective: estimate = <0.0001, Pp = 0, Hz change = 0]. The direct comparison thus shows a down‐regulation of these connections after effective TMS.

Concerning intrinsic connectivity between two areas, TMS lowered the inhibition from the right cerebellum on the left SMG [sham: estimate = −0.130, Pp = 1; effective: estimate = <0.0001, Pp = 0]. Moreover, TMS caused an inhibition of the left SMG on the right vOTC [sham: estimate = <0.0001, Pp = 0; effective: estimate = −0.086, Pp = 1]. Also, the nonsignificant connection from the left to the right vOTC during sham was significant after effective TMS [sham: estimate = <0.0001, Pp = 0; effective: estimate = 0.058, Pp = 0.947]. A visual comparison of the sham and effective TMS DCM illustrates the strong and manifold modulations of the triangle among the left vOTC, the right vOTC, and the right cerebellum. After effective TMS, we saw reduced modulations in this triangle, which are supported by the direct comparison of DCMs (effective vs. sham). Overall, our DCM results suggest stronger interactions from the left SMG for pseudowords and reduced modulations in the triangle involving the left and right vOTC and the right cerebellum.

### Differential activation of the left TPC subregions during word and pseudoword reading

**FIGURE 5:**
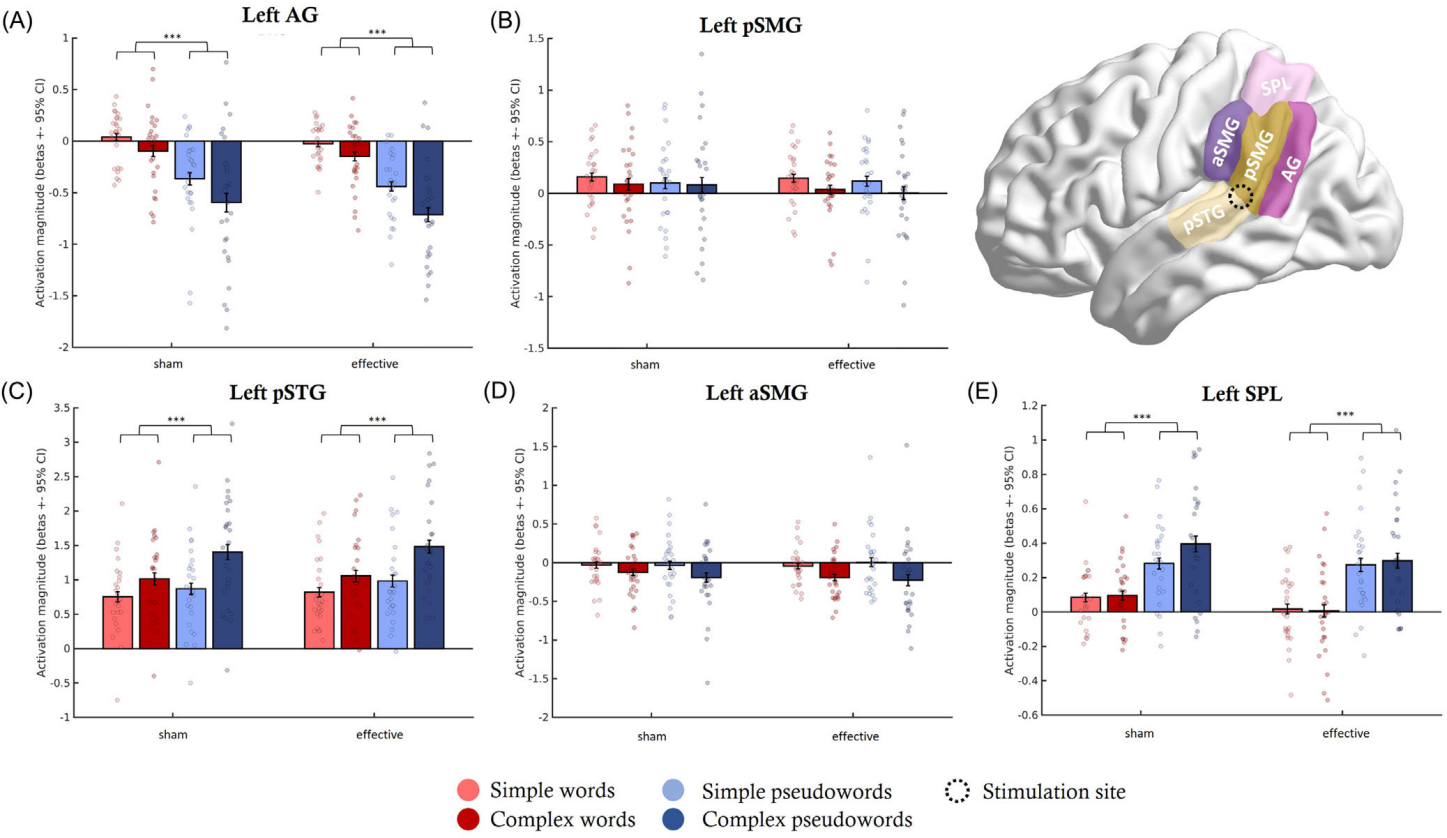
Activation magnitude for subregions within the left TPC (the left AG, pSMG, pSTG, aSMG, and SPL) for the four conditions (simple words, complex words, simple pseudowords, and complex pseudowords) during both effective and sham stimulations. The stimulation site is marked in the illustration in the right top corner. Abbreviations: AG, angular gyrus; a/pSMG, anterior/posterior supramarginal gyrus; pSTG, posterior superior temporal gyrus; SPL, superior parietal lobe.

As the left TPC is a crucial area for phonological decoding and covers a large range of adjacent regions, we additionally explored which region within this area contributed to the processing of the two stimulus types and their complexity, and if these effects interacted with TMS. Therefore, we investigated activation magnitude in the form of beta values derived from the effective and sham TMS‐fMRI sessions from five subregions of the left TPC (AG, the left aSMG, pSMG, pSTG, and SPL). The exact locations of these regions based on the Harvard‐Oxford atlas as well as the stimulation site at the border between pSTG and pSMG are shown in Figure 5. Note that, while all results are discussed below, only significant main effects of stimulus type are visually displayed.

Confirming our functional activation analyses, no region within the left TPC showed a main effect of TMS. However, the left AG, pSTG, and SPL showed significant, differential engagements depending on whether the subjects read words or pseudowords. The left AG showed both a significant main effect for stimulus type (F_(1,25)_ = 55.38, *p* < 0.001, *η* ^2^ = 0.398) and a weak main effect of complexity (F_(1,25)_ = 32.11, *p* < 0.001, *η* ^2^ = 0.065). Specifically, left AG showed significantly stronger deactivation for pseudowords than words, and for complex than simple stimuli. The left pSTG also showed the main effects of stimulus type (F_(1,25)_ = 42.81, *p* < 0.001, *η* ^2^ = 0.138) and complexity (F_(1,25)_ = 83, *p* < 0.001, *η* ^2^ = 0.270). As opposed to the left AG, the left pSTG showed higher functional activation for pseudowords than words, and for complex than simple stimuli. Furthermore, the interaction between complexity and stimulus type was significant (F_(1,25)_ = 37.78, *p* < 0.001, *η* ^2^ = 0.033), with significant differences between simple words and complex words (*t* = −5.262, *p* < 0.001), simple words and simple pseudowords (*t* = −2.962, *p* < 0.001), complex words and complex pseudowords (*t* = −8.642, *p* < 0.001), and simple words and complex pseudowords (*t* = −11.074, *p* < 0.001). Note that this effect was weak (*η* ^2^ < 0.052) given our current sample size and should thus be interpreted with caution. Similarly, the left SPL showed higher activation for pseudowords as compared to words (F_(1,25)_ = 87.70, *p* < 0.001, *η* ^2^ = 0.357).

Regarding the two subdivisions of the left SMG, the left aSMG showed differential engagement for simple and complex stimuli (F _(1,25)_ = 38.26, *p* < 0.001, *η* ^2^ = 0.109). Post‐hoc tests showed significant differences in recruitment between simple and complex words (*t* = 3.960, *p* = 0.001; higher deactivation for complex words), simple and complex pseudowords (*t* = 6.337, *p* < 0.001; higher deactivation for complex pseudowords), as well as simple pseudowords and complex words (*t* = −2.742, *p* = 0.027) and simple words and complex pseudowords (*t* = 3.239, *p* = 0.01). Although we also found the same effect in the left pSMG (F_(1,25)_ = 7.74, *p* = 0.01, *η* ^2^ = 0.010), this effect should be interpreted with caution as we did not have enough power to reliably detect this effect. Overall, it seems that more complex stimuli could be linked to less activation in the left pSMG and higher deactivation in the left aSMG.

To summarize, pseudowords selectively engaged the left pSTG and the left SPL more than words and were characterized by stronger deactivation in the left AG. Words, in contrast, showed less deactivation in the left AG but did not engage any subregion of the left TPC more than pseudowords. Looking at the processing of complexity, both the left SMG regions and the left AG deactivated for complex items, whereas the left pSTG showed higher activation for complex items.

## DISCUSSION

The present study explored TMS‐induced effects on reading performance, functional activation, and connectivity within reading networks in adults with dyslexia. At the behavioral level, facilitation of the left TPC improved speech onsets for complex pseudowords, and faster reading was directly linked to functional coupling between the left IFG and the left vOTC. Stimulation led to a lower recruitment of right‐hemispheric areas and significantly altered neural interactions in classical and extended, hypoactive reading networks. In addition to stronger recruitment of the ventral reading route, stronger pseudoword‐specific connectivity from the left SMG and lower inhibition from the right cerebellum on reading areas could play a role in improving phonological decoding in dyslexia. Moreover, only the aSMG showed atypical recruitment for pseudowords, suggesting a causal role for phonological deficits in dyslexia.

TMS‐induced facilitation of the left TPC significantly improved reading performance, especially speech onsets of complex pseudowords. Both reading times and accuracies for complex pseudowords showed strong learning effects, making it difficult to disentangle TMS and reading effects on these two parameters despite highly significant interactions. Participants who received effective stimulation in their first session had better performance already during the first session, but still improved due to learning/session effects, again resulting in better performance. These results emphasize the strong impact of baseline performance on the modulatory potential of TMS.^86^ For therapeutic applications, this suggests that participants who benefit less from reading interventions alone or are more severely affected would be more sensitive to adjuvant treatment with TMS. Based on our results, we reason that the left TPC is a promising candidate for neuromodulatory interventions. We thus corroborate the findings of the few behavioral studies to date which showed that left TPC stimulation improves reading performance in children and adults with dyslexia.^32^, ^35^, ^36^, ^38^, ^58^ Interestingly, we only found effects for complex and not simple pseudowords, which might indicate that more challenging phonological decoding tasks are required to reveal subtle TMS effects. We already indicated in earlier work that simple pseudoword reading might be too easy to observe differences when comparing individuals with and without dyslexia.^26^ Whereas both speech onsets and overt reading times can be seen as reading processes, we believe that the latter is more influenced by other factors (e.g., personal reading speed, nervousness, and anxiety) and reflects speech articulation more than reading. Therefore, we interpret speech onsets as a better measure of reading as it reflects the inner process of putting together sounds and letters before starting the articulation process, or, in the case of words, is a measure of lexical access.

Facilitation of the left TPC decreased right‐hemispheric contributions to reading. Contrary to our hypotheses, we found no stimulation‐induced changes in task‐related activity at the stimulation site. Nevertheless, the present study provides the first evidence that iTBS to a key reading node decreases the task‐specific contribution of right‐hemispheric regions. TMS decreased task‐related activity in the right precuneus/bIPS during word reading and in the right insula/auditory cortex during pseudoword reading. These areas are not part of the classical reading network, and their recruitment may reflect demand‐related processes. The difference in the observed stimulation effects for words and pseudowords suggests that these regions were recruited in a task‐specific fashion. Earlier studies linked the right IPS to various cognitive functions, especially arithmetic,^87^, ^88^ visuo‐spatial working memory,^89^ and attention.^90^ Similarly, the bilateral precuneus has been shown to be engaged during reading due to semantic demands,^91^ but most focus has been on the left precuneus, which shows structural changes after reading training.^92^ However, there is evidence that the right anterior IPS engages more strongly in individuals with dyslexia^93^ and that there is a negative correlation between right‐hemispheric brain activity and reading ability.^94^ Therefore, it seems likely that facilitation in the left TPC, which leads to better reading performance, decreases the contribution of regions that were potentially more engaged beforehand. Recently, it has been suggested that gray matter correlates of reading and attention overlap in the left precuneus, which may point toward a shared neural substrate for the comorbidity of dyslexia and attention‐deficit/hyperactivity disorder.^95^ Since we excluded subjects with attention deficits, our study does not corroborate these findings but rather suggests a reading‐ or automaticity‐specific recruitment of the right precuneus. The observed shift in the right insula during pseudoword reading seems to be of a similar nature. Right anterior insula activation has been linked to cognitive load and linguistic effort,^96^ which explains why activation could be linked to pseudoword processing, which has a higher difficulty than word reading. Moreover, the right insula was tied to behavioral reading performance in children with dyslexia,^97^ and its anterior portion shows reduced response to various presentation modalities in dyslexia.^98^

Stimulation‐induced facilitation of the left TPC changed effective connectivity within classical and extended reading networks. Specifically, facilitation of the left TPC led to significant changes in the reading network architecture, from a stronger modulation of the interaction along the ventral route (IFG—vOTC) to stronger modulations from the left SMG to the left vOTC and the right cerebellum, vital areas for pseudoword reading. Crucially, the significantly stronger modulation of the left IFG‐to‐vOTC coupling after effective stimulation correlated with decreased speech onsets for complex pseudowords, showing a direct link between neural and behavioral changes. These results are congruent with earlier findings that reported the behavioral relevance of remote stimulation effects in healthy adults, even in the absence of direct effects at the stimulation site.^99^ Together, the previous and present results support the notion that TMS modulates network interactions rather than focal activity at the stimulated area. Consequently, targeting densely connected hub regions may be promising for the treatment of network disorders, including dyslexia.

We further highlight that regions within the left TPC show stimulus‐ and complexity‐specific activation in dyslexia. The left TPC region has been deemed crucial for dyslexia^100^ due to reports of its hypoactivation in dyslexia.^14^, ^16^, ^18^ Here, only three regions within the broader TPC area showed differential recruitment for word and pseudoword reading. While the left AG deactivated more for pseudowords, we found higher activation in the left pSTG and the left SPL for pseudoword reading, consistent with previous work.^101^ In a recent comparison of typical readers and adults with dyslexia, the hypoactive SMG was identified as a region responding mainly to pseudowords in typical readers, while adults with dyslexia lacked this preferential engagement.^26^ Specifically, the left aSMG, SPL, and pSTG regions showed higher activation for pseudowords than words in typical readers.^39^ Based on our current findings, we provide strong evidence for a lack of specialization for phonological decoding in the left aSMG. Preferential recruitment of the left pSTG and SPL was also found in typical readers,^39^ suggesting that only the left aSMG shows atypical engagement during reading in dyslexia. Previous studies have highlighted individual changes in brain activation in the left pSTG and adjacent regions after remediation,^102^ indicating that a preferential engagement of this area might be crucial for pseudoword reading. We may thus speculate that this area is a promising candidate for individualized stimulation when phonological deficits are prominent.

Overall, our findings suggest that communications among the left SMG, the left vOTC, and the right cerebellum present crucial elements in the neural architecture underlying reading in dyslexia. In an earlier study, pseudoword‐specific hypoactivation in dyslexia was mainly found in the left vOTC and the right cerebellum.^26^ Interestingly, a DCM involving these two regions and two other regions that are usually hypoactive in dyslexia revealed that facilitation of the left TPC resulted in stronger effective connectivity from the left SMG to the left vOTC and from the left SMG to the right cerebellum during pseudoword reading only. Generally, we also observed stronger functional coupling for overall reading between the right cerebellum and the left SMG, as well as the left vOTC and the right vOTC. Interestingly, however, the interaction between the right vOTC and the right cerebellum for all stimuli was decreased after iTBS to left TPC. At the same time, the right vOTC showed a down‐regulation (lower self‐inhibition) after effective TMS.

Taken together, these findings confirm that neural network interactions in reading circuits are crucial for dyslexia and TMS has the potential to induce changes that lead to improved reading performance. Despite a lack of changes in functional activation in reading areas after effective stimulation, our results support a close interaction between reading areas, much in line with connectionist models of reading.^103^ Supporting earlier studies, we suggest that the dorsal and ventral reading streams^104^ are vital for pseudoword reading in adults with dyslexia and might undergo short‐term changes after neurostimulation to modulate reading. We highlight the need to consider TMS‐induced effects on the network level, even in the absence of a modulatory effect in the stimulated areas, since TMS may rather lead to large‐scale changes on the network level that are reflected in remote activity within targeted or other networks.^105^ We reason that similar large‐scale network effects should also occur for other neurostimulation techniques such as transcranial electric stimulation. Indeed, previous studies combining functional neuroimaging and tDCS also reported remote effects in connected neighboring or distant areas of the targeted network for different language tasks.^106^, ^107^ However, we are not aware of any study directly comparing the modulatory effects of TMS and tDCS within the same participants and paradigm. Moreover, while some of the previous tDCS studies also reported distributed network effects, most of them found modulatory changes in the targeted area. Based on the present findings, it might be necessary to extend current models of reading and more closely investigate the contributions of the right cerebellum and the engagement of the ventral reading route during word and pseudoword reading in dyslexia.

We would like to add that the present study does have limitations. First, we only included a single session of TMS, which is certainly not as powerful as repeated stimulation. Second, we selectively included placebo stimulation as the control for our effective stimulation. The placebo coil mimics stimulation noise but does not deliver effective stimulation, which might make it easier for some subjects with stronger side effects to notice the difference. We note that including both a sham TMS baseline and an active control site is generally preferable, as it provides the highest level of control.^108^ However, in studies of higher cognition, it can be tricky to identify areas that are not linked to task processing at all. We explicitly chose sham stimulation instead of an active control site as we wanted to avoid targeting any area within networks relevant to the task (e.g., cognitive control or attention networks). Third, our stimulation site was not motivated by individual activation patterns or a localizer, which might be helpful in increasing stimulation efficiency in the future. Fourth, a direct comparison with typical readers (i.e., using the same stimulation protocol and task) would allow stronger statements regarding the specificity of the observed effects in the dyslexia population. In our recent study, we applied inhibitory stimulation in an age‐matched typical reading group^39^ as we aimed to first probe the relevance of the TPC for reading in the healthy reading network. In that study, we reasoned that task performance might be at the ceiling for healthy controls. Future studies may include more challenging reading conditions (i.e., by including even more complex pseudowords with a higher number of syllables) to test whether performance can also be improved in healthy volunteers with iTBS or transcranial electrical stimulation. Last, a natural reading paradigm would be more suitable to discuss activation during reading. However, to reliably investigate TMS effects, a well‐controlled paradigm is needed. Therefore, we decided to go for overt word and pseudoword reading, which allowed us to closely investigate the speed and accuracy of reading.

## CONCLUSION

Here, we show that facilitating a core reading node can successfully improve phonological decoding in adults with dyslexia. In addition to improvements in complex pseudoword reading, neurostimulation decreased the contribution of the right hemisphere and altered connectivity between core reading nodes and the right cerebellum. These neural findings were directly linked to behavioral improvements. Our results increase the understanding of neuromodulatory interventions for individuals with dyslexia and elucidate the immediate plastic after‐effects of neurostimulation on the reading network. This, in turn, is crucial for advancing targeted network stimulation for future treatment purposes in dyslexia. Our results show that neurostimulation provides a powerful means to modulate network interactions and change connectivity in remote parts of the network. These findings indicate that potential improvements after neurostimulation may not (only) be caused by direct modulatory changes at the stimulated area, but rather at the network level, which is in line with previous TMS studies in healthy volunteers (e.g., Refs. 99 and 109). We note that such network changes cannot be detected by pure behavioral studies, demonstrating the power of combined TMS‐fMRI to modulate and map the impact of neurostimulation at the neural network and behavioral level. With respect to therapeutic applications in participants with dyslexia or other network disorders, our findings suggest that targeting densely connected hub areas within a network of interest may be a way forward to reach distributed parts of specialized networks and improve stimulation efficiency.

## AUTHOR CONTRIBUTIONS

S.T.: Study design, data collection, data analysis, and manuscript writing; P.K.: Data analysis and editing the manuscript; V.K.M.C.: Data analysis; K.W.: Data analysis; and G.H.: Study design and editing the manuscript.

## COMPETING INTERESTS

The authors declare no competing financial or nonfinancial interests.

### PEER REVIEW

The peer review history for this article is available at: https://publons.com/publon/10.1111/nyas.15291

## Supporting information




**Table S1**. Summary of behavioral and demographic information.
**Table S2**. Post‐hoc tests for significant TMS interactions for reading times: (1) TMS by stimulus type, (2) TMS by complexity by stimulus type and (3) TMS by complexity by stimulus type by session.
**Table S3**. Post‐hoc tests for significant TMS interactions for accuracy: (1) TMS by session, (2) TMS by complexity by stimulus type and (3) TMS by complexity by stimulus type by session.
**Table S4**. DCM results (parameter estimates, posterior probability, and Hz change for stimulus‐specific modulations) for the classical reading network DCM (left pSTG, left IFG, left vOTC) for the sham condition.
**Table S5**. DCM results (parameter estimates, posterior probability, and Hz change for stimulus‐specific modulations) for the classical reading network DCM (left pSTG, left IFG, left vOTC) for the effective condition.
**Table S6**. DCM results for the classical reading network DCM (left pSTG, left IFG, left vOTC) for the effective versus sham condition.
**Table S7**. DCM results (parameter estimates, posterior probability, and Hz change for stimulus‐specific modulations) for the extended reading network (left SMG, left vOTC, right vOTC, right cerebellum) for the sham condition.
**Table S8**. DCM results (parameter estimates, posterior probability, and Hz change for stimulus‐specific modulations) for the extended reading network (left SMG, left vOTC, right vOTC, right cerebellum) for the effective condition.
**Table S9**. DCM results for the extended reading network (left SMG, left vOTC, right vOTC, right cerebellum) for the effective versus sham condition. Positive and negative values indicate the significant findings for the contrast of conditions: effective > sham (0 to 1; positive) and sham > effective (0 to −1; negative).
**Figure S1**. Individual e‐field simulation results and average e‐field and standard deviation. Colors indicate the electrical field in V/m.
