# Supplementary material for: Neurostimulation improves reading and alters communication within reading networks in dyslexia
Source: Ann N Y Acad Sci. 2025 Feb 1;1544(1):172–89. doi: 10.1111/nyas.15291 (PMC11829325; doi:10.1111/nyas.15291)
Supplement: Supplementary file 1 — Table S1. Summary of behavioral and demographic information. Table S2. Post‐hoc tests for significant TMS interactions for reading times: (1) TMS by stimulus type, (2) TMS by complexity by stimulus type and (3) TMS by complexity by stimulus type by session. Table S3. Post‐hoc tests for significant TMS interactions for accuracy: (1) TMS by session, (2) TMS by complexity by stimulus type and (3) TMS by complexity by stimulus type by session. Table S4. DCM results (parameter estimates, posterior probability, and Hz change for stimulus‐specific modulations) for the classical reading network DCM (left pSTG, left IFG, left vOTC) for the sham condition. Table S5. DCM results (parameter estimates, posterior probability, and Hz change for stimulus‐specific modulations) for the classical reading network DCM (left pSTG, left IFG, left vOTC) for the effective condition. Table S6. DCM results for the classical reading network DCM (left pSTG, left IFG, left vOTC) for the effective versus sham condition. Table S7. DCM results (parameter estimates, posterior probability, and Hz change for stimulus‐specific modulations) for the extended reading network (left SMG, left vOTC, right vOTC, right cerebellum) for the sham condition. Table S8. DCM results (parameter estimates, posterior probability, and Hz change for stimulus‐specific modulations) for the extended reading network (left SMG, left vOTC, right vOTC, right cerebellum) for the effective condition. Table S9. DCM results for the extended reading network (left SMG, left vOTC, right vOTC, right cerebellum) for the effective versus sham condition. Positive and negative values indicate the significant findings for the contrast of conditions: effective > sham (0 to 1; positive) and sham > effective (0 to −1; negative). Figure S1. Individual e‐field simulation results and average e‐field and standard deviation. Colors indicate the electrical field in V/m. [file NYAS-1544-172-s001.docx]

**Supplementary Materials**

**Supplementary Table S1.** Summary of behavioural and demographic information. Information on values is provided in brackets (s = seconds). Please note that for accuracy scores on in-scanner performance, each trial with an error (e.g., an omitted letter or a substituted vowel) was counted as an incorrect response and might thus lead to particularly low scores for complex pseudowords, which were most challenging for participants.

|  | **Demographics** | **mean (std)** | **min - max** |
| --- | --- | --- | --- |
|  | Age (years) | 26.5 (5.9) | 18 - 39 |
|  | Word reading (number of words) | 76 (17.8) | 49 - 107 |
|  | Nonword reading (number of nonwords) | 45.9 (17.1) | 22 - 98 |
|  | Text comprehension (T value) | 45.9 (7.5) | 30 - 61 |
|  | Text reading speed (T value) | 40.4 (8.3) | 27 - 57 |
|  | Text reading accuracy (T value) | 60 (10.5) | 34 - 73 |
|  | Spelling (number of errors) | 35.5 (10.1) | 17 - 50 |
|  | Spoonerisms (mean production time) | 7.4 (3.5) | 2.6 - 16.1 |
|  | Digit span forward | 7.1 (1.9) | 3 - 11 |
|  | Digit span backward | 5.9 (1.9) | 2 - 10 |
|  | Nonword span (number of syllables) | 116.8 (32) | 45 – 181 |
| **Condition** | **In-scanner performance** |  |  |
| **sham** | Speech onsets simple words (s) | 0.91 (0.17) | 0.66-1.27 |
|  | Speech onsets simple pseudowords (s) | 1.13 (0.24) | 0.77-1.76 |
|  | Speech onsets complex words (s) | 1.04 (0.21) | 0.73-1.54 |
|  | Speech onsets complex pseudowords (s) | 1.27 (0.19) | 0.88-1.82 |
|  | Overt reading simple words (s) | 0.58 (0.08) | 0.43-0.78 |
|  | Overt reading simple pseudowords (s) | 0.68 (0.10) | 0.45-0.90 |
|  | Overt reading complex words (s) | 0.94 (0.12) | 0.71-1.21 |
|  | Overt reading complex pseudowords (s) | 1.69 (0.22) | 1.24-2.14 |
|  | Accuracy simple words (%) | 99.1 (2.1) | 90-100 |
|  | Accuracy simple pseudowords (%) | 92.9 (7.5) | 72-100 |
|  | Accuracy complex words (%) | 98.2 (2.4) | 91.7-100 |
|  | Accuracy complex pseudowords (%) | 67.4 (18) | 28-96 |
| **effective** | Speech onsets simple words (s) | 0.89 (0.16) | 0.67-1.31 |
|  | Speech onsets simple pseudowords (s) | 1.11 (0.23) | 0.79-1.79 |
|  | Speech onsets complex words (s) | 1 (0.19) | 0.74-1.62 |
|  | Speech onsets complex pseudowords (s) | 1.22 (0.19) | 0.83-1.68 |
|  | Overt reading simple words (s) | 0.57 (0.06) | 0.41-0.73 |
|  | Overt reading simple pseudowords (s) | 0.68 (0.07) | 0.50-0.86 |
|  | Overt reading complex words (s) | 0.95 (0.12) | 0.74-1.17 |
|  | Overt reading complex pseudowords (s) | 1.70 (0.20) | 1.40-2.21 |
|  | Accuracy simple words (%) | 99.4 (1.2) | 95.9-100 |
|  | Accuracy simple pseudowords (%) | 93.5 (5.7) | 74-100 |
|  | Accuracy complex words (%) | 97.9 (4) | 85.4-100 |
|  | Accuracy complex pseudowords (%) | 68.5 (19) | 16-92 |

**Supplementary Table S2.** Post-hoc tests for significant TMS interactions for reading times: (1) TMS by stimulus type, (2) TMS by complexity by stimulus type and (3) TMS by complexity by stimulus type by session.

| **Interaction** | **estimate** | **t-ratio** | **p-value** |
| --- | --- | --- | --- |
| **TMS: Stimulus type** |  |  |  |
| Words (active vs. sham) | -0.000 | -0.037 | 0.970 |
| Pseudowords (active vs. sham) | -0.004 | -0.340 | 0.734 |
| **TMS : Complexity : Stimulus type** |  |  |  |
| Simple words (active vs sham) | -0.011 | -1.226 | 0.220 |
| Complex words (active vs sham) | 0.011 | 0.984 | 0.325 |
| Simple pseudowords (active vs sham) | -0.004 | -0.404 | 0.686 |
| Complex pseudowords (active vs sham) | -0.004 | -0.223 | 0.823 |
| **TMS : Complexity : Stimulus type: Session** |  |  |  |
| Simple words (active – sham) | -0.014 | -0.913 | 0.361 |
| Complex words (active – sham) | 0.020 | 1.300 | 0.194 |
| Simple pseudowords (active – sham) | 0.013 | 0.707 | 0.480 |
| Complex pseudowords (active – sham) | 0.147 | 6.301 | <0.001*** |
| Simple words (sham - active) | -0.009 | -0.480 | 0.619 |
| Complex words (sham – active) | -0.027 | -1.465 | 0.143 |
| Simple pseudowords (sham – active) | 0.009 | 0.404 | 0.686 |
| Complex pseudowords (sham – active) | -0.154 | -5.317 | <0.001*** |

***: p < 0.001

**Supplementary Table S3.** Post-hoc tests for significant TMS interactions for accuracy: (1) TMS by session, (2) TMS by complexity by stimulus type and (3) TMS by complexity by stimulus type by session.

| **Interaction** | **Odd’s ratio** | **z-ratio** | **p-value** |
| --- | --- | --- | --- |
| **TMS: Session** |  |  |  |
| Active - sham | 0.711 | -1.249 | 0.212 |
| Sham - active | 1.984 | 1.994 | 0.046* |
| **TMS: Complexity: Session** |  |  |  |
| Simple items (active – sham) | 0.622 | -1.048 | 0.295 |
| Simple items (sham – active) | 3.309 | 2.056 | 0.040* |
| Complex items (active – sham) | 0.812 | -0.805 | 0.421 |
| Complex items (sham – active) | 1.190 | 0.596 | 0.551 |
| **TMS: Stimulus type: Session** |  |  |  |
| Words (active – sham) | 0.554 | -1.228 | 0.219 |
| Words (sham – active) | 2.978 | 1.808 | 0.070 |
| Pseudowords (active – sham) | 0.912 | -0.488 | 0.626 |
| Pseudowords (sham – active) | 1.322 | 1.217 | 0.224 |
| **TMS : Complexity : Stimulus Type: Session** |  |  |  |
| Simple words (active – sham) | 0.351 | -1.246 | 0.213 |
| Complex words (active – sham) | 1.103 | -0.301 | 0.726 |
| Simple pseudowords (active – sham) | 0.873 | -0.301 | 0.763 |
| Complex pseudowords (active – sham) | 0.755 | -1.503 | 0.133 |
| Simple words (sham - active) | 10.655 | 2.175 | 0.030* |
| Complex words (sham – active) | 1.028 | 0.084 | 0.933 |
| Simple pseudowords (sham – active) | 0.832 | -0.379 | 0.704 |
| Complex pseudowords (sham – active) | 1.701 | 2.217 | 0.027* |

*: p < 0.05

**Supplementary Table S4**. DCM results (parameter estimates, posterior probability and Hz change for stimulus-specific modulations) for the classical reading network DCM (left pSTG, left IFG, left vOTC) for the sham condition.

| **Connection** | **Parameter estimate** | **Posterior Probability** | **Hz change** |
| --- | --- | --- | --- |
| L pSTG to L pSTG | -0.291 | 1 |  |
| L pSTG to L IFG | 0.107 | 1 |  |
| L pSTG to L vOTC | 0.136 | 1 |  |
| L IFG to L pSTG | -0.127 | 1 |  |
| L IFG to L IFG | -0.148 | 1 |  |
| L IFG to L vOTC | -0.198 | 1 |  |
| L vOTC to L pSTG | -0.122 | 0.999 |  |
| L vOTC to L IFG | -0.104 | 0.999 |  |
| L vOTC to L vOTC | -0.354 | 1 |  |
| L pSTG to L IFG (words) | 0.000 | 0 | 0.050 |
| L IFG to L pSTG (words) | -0.413 | 0.982 | -0.207 |
| L IFG to L vOTC (words) | 0.832 | 1 | 0.439 |
| L vOTC to L pSTG (words) | 0.462 | 0.989 | 0.134 |
| L vOTC to L pSTG (words) | 0.659 | 1 | 0.338 |
| L vOTC to L IFG (words) | 0.000 | 0 | -0.146 |
| L pSTG to L IFG (pseudowords) | 0.332 | 0.959 | 0.382 |
| L pSTG to L vOTC (pseudowords) | 0.000 | 0 | 0.207 |
| L IFG to L pSTG (pseudowords | 0.725 | 1 | 0.332 |
| L IFG to L vOTC (pseudowords) | 0.295 | 0.904 | -0.033 |
| L vOTC to L pSTG (pseudowords) | 0.506 | 1 | 0.185 |
| L vOTC to L IFG (pseudowords) | 0.246 | 0.864 | 0.100 |
| L pSTG (all trials) | 0.594 | 1 |  |
| L IFG (all trials) | 0.365 | 1 |  |
| L vOTC (all trials) | 0.413 | 1 |  |

**Supplementary Table S5**. DCM results (parameter estimates, posterior probability and Hz change for stimulus-specific modulations) for the classical reading network DCM (left pSTG, left IFG, left vOTC) for the effective condition.

| **Connection** | **Parameter estimate** | **Posterior Probability** | **Hz change** |
| --- | --- | --- | --- |
| L pSTG to L pSTG | -0.460 | 1 |  |
| L pSTG to L IFG | 0.132 | 1 |  |
| L pSTG to L vOTC | 0.042 | 0.674 |  |
| L IFG to L pSTG | 0.000 | 0 |  |
| L IFG to L IFG | -0.110 | 0.979 |  |
| L IFG to L vOTC | -0.158 | 1 |  |
| L vOTC to L pSTG | -0.293 | 1 |  |
| L vOTC to L IFG | -0.152 | 1 |  |
| L vOTC to L vOTC | -0.179 | 1 |  |
| L pSTG to L IFG (words) | 0.000 | 0 | 0.090 |
| L IFG to L pSTG (words) | -0.305 | 0.947 | -0.192 |
| L IFG to L vOTC (words) | 0.681 | 1 | 0.388 |
| L vOTC to L pSTG (words) | 0.398 | 0.970 | -0.001 |
| L vOTC to L pSTG (words) | 0.293 | 0.946 | -0.138 |
| L vOTC to L IFG (words) | 0.000 | 0 | -0.204 |
| L pSTG to L IFG (pseudowords) | 0.252 | 0.907 | 0.341 |
| L pSTG to L vOTC (pseudowords) | -0.116 | 0.511 | -0.003 |
| L IFG to L pSTG (pseudowords | 1.037 | 1 | 0.744 |
| L IFG to L vOTC (pseudowords) | 1.018 | 1 | 0.619 |
| L vOTC to L pSTG (pseudowords) | 0.513 | 1 | 0.082 |
| L vOTC to L IFG (pseudowords) | 0.308 | 0.978 | 0.103 |
| L pSTG (all trials) | 0.497 | 1 |  |
| L IFG (all trials) | 0.352 | 1 |  |
| L vOTC (all trials) | 0.436 | 1 |  |

**Supplementary Table S6**. DCM results for the classical reading network DCM (left pSTG, left IFG, left vOTC) for the effective vs. sham condition. Positive and negative values indicate the significant findings for the contrast of conditions: effective > sham (0 to 1; positive) and sham > effective (0 to -1; negative).

| **Connection** | **Parameter estimate** | **Posterior Probability** |
| --- | --- | --- |
| L pSTG to L pSTG | -0.069 | 0.874 |
| L pSTG to L IFG | 0.000 | 0 |
| L pSTG to L vOTC | -0.027 | 0.543 |
| L IFG to L pSTG | 0.000 | 0 |
| L IFG to L IFG | 0.000 | 0 |
| L IFG to L vOTC | 0.000 | 0 |
| L vOTC to L pSTG | -0.064 | 0.848 |
| L vOTC to L IFG | 0.000 | 0 |
| L vOTC to L vOTC | 0.060 | 0.868 |
| L pSTG to L IFG (words) | 0.000 | 0 |
| L IFG to L pSTG (words) | 0.000 | 0 |
| L IFG to L vOTC (words) | 0.000 | 0 |
| L vOTC to L pSTG (words) | 0.000 | 0 |
| L vOTC to L pSTG (words) | 0.000 | 0 |
| L vOTC to L IFG (words) | 0.000 | 0 |
| L pSTG to L IFG (pseudowords) | 0.000 | 0 |
| L pSTG to L vOTC (pseudowords) | -0.109 | 0.527 |
| L IFG to L pSTG (pseudowords | 0.000 | 0 |
| L IFG to L vOTC (pseudowords) | 0.402 | 1 |
| L vOTC to L pSTG (pseudowords) | 0.000 | 0 |
| L vOTC to L IFG (pseudowords) | 0.000 | 0 |
| L pSTG (all trials) | 0.000 | 0 |
| L IFG (all trials) | 0.000 | 0 |
| L vOTC (all trials) | 0.000 | 0 |

**Supplementary Table S7**. DCM results (parameter estimates, posterior probability and Hz change for stimulus-specific modulations) for the extended reading network (left SMG, left vOTC, right vOTC, right cerebellum) for the sham condition.

| **Connection** | **Parameter estimate** | **Posterior probability** | **Hz change** |
| --- | --- | --- | --- |
| L SMG to L SMG | -0.603 | 1 |  |
| L SMG to L vOTC | -0.119 | 1 |  |
| L SMG to R Cerebellum | 0.000 | 0 |  |
| L SMG to R vOTC | 0.000 | 0 |  |
| L vOTC to L SMG | 0.111 | 1 |  |
| L vOTC to L vOTC | -0.562 | 1 |  |
| L vOTC to R Cerebellum | 0.054 | 0.964 |  |
| L vOTC to R vOTC | 0.000 | 0 |  |
| R Cerebellum to L SMG | -0.130 | 1 |  |
| R Cerebellum to L vOTC | -0.162 | 1 |  |
| R Cerebellum to R Cerebellum | -0.516 | 1 |  |
| R Cerebellum to R vOTC | -0.092 | 1 |  |
| R vOTC to L SMG | 0.000 | 0 |  |
| R vOTC to L vOTC | -0.078 | 0.948 |  |
| R vOTC to R Cerebellum | 0.000 | 0 |  |
| R vOTC to R vOTC | -0.413 | 1 |  |
| L SMG to L vOTC (words) | 0.697 | 1 | 0.459 |
| L SMG to R Cerebellum (words) | 0.000 | 0 | 0.000 |
| L SMG to R vOTC (words) | 0.536 | 1 | 0.444 |
| L vOTC to L SMG (words) | 0.000 | 0 | 0.064 |
| L vOTC to R Cerebellum (words) | 0.000 | 0 | 0.054 |
| L vOTC to R vOTC (words) | 0.378 | 1 | 0.259 |
| R Cerebellum to L SMG (words) | 0.000 | 0 | -0.174 |
| R Cerebellum to L vOTC (words) | 0.000 | 0 | -0.264 |
| R Cerebellum to R vOTC (words) | 0.000 | 0 | -0.177 |
| R vOTC to L SMG (words) | 0.000 | 0 | 0.000 |
| R vOTC to L vOTC (words) | 0.308 | 0.953 | 0.083 |
| R vOTC to R Cerebellum (words) | 0.327 | 0.994 | 0.195 |
| L SMG to L vOTC (pseudowords) | 0.000 | 0 | -0.238 |
| L SMG to R Cerebellum (pseudowords) | 0.000 | 0 | 0.000 |
| L SMG to R vOTC (pseudowords) | 0.000 | 0 | -0.091 |
| L vOTC to L SMG (pseudowords) | 0.272 | 0.914 | 0.337 |
| L vOTC to R Cerebellum (pseudowords) | 0.000 | 0 | 0.054 |
| L vOTC to R vOTC (pseudowords) | 0.316 | 0.925 | 0.198 |
| R Cerebellum to L SMG (pseudowords) | 0.260 | 0.799 | 0.086 |
| R Cerebellum to L vOTC (pseudowords) | 0.598 | 1 | 0.334 |
| R Cerebellum to R vOTC (pseudowords) | 0.496 | 0.990 | 0.319 |
| R vOTC to L SMG (pseudowords) | 0.000 | 0 | 0.000 |
| R vOTC to L vOTC (pseudowords) | 0.545 | 1 | 0.319 |
| R vOTC to R Cerebellum (pseudowords) | 0.443 | 1 | 0.312 |
| L SMG (all trials) | 0.000 | 0 |  |
| L vOTC (all trials) | 0.312 | 1 |  |
| R Cerbellum (all trials) | 0.000 | 0 |  |
| R vOTC (all trials) | 0.257 | 1 |  |

**Supplementary Table S8**. DCM results (parameter estimates, posterior probability and Hz change for stimulus-specific modulations) for the extended reading network (left SMG, left vOTC, right vOTC, right cerebellum) for the effective condition.

| **Connection** | **Parameter estimate** | **Posterior probability** | **Hz change** |
| --- | --- | --- | --- |
| L SMG to L SMG | -0.522 | 1 |  |
| L SMG to L vOTC | -0.123 | 1 |  |
| L SMG to R Cerebellum | -0.059 | 1 |  |
| L SMG to R vOTC | -0.086 | 1 |  |
| L vOTC to L SMG | 0.070 | 1 |  |
| L vOTC to L vOTC | -0.494 | 1 |  |
| L vOTC to R Cerebellum | 0.097 | 1 |  |
| L vOTC to R vOTC | 0.058 | 0.947 |  |
| R Cerebellum to L SMG | 0.000 | 0 |  |
| R Cerebellum to L vOTC | -0.117 | 1 |  |
| R Cerebellum to R Cerebellum | -0.614 | 1 |  |
| R Cerebellum to R vOTC | 0.073 | 1 |  |
| R vOTC to L SMG | 0.000 | 0 |  |
| R vOTC to L vOTC | 0.000 | 0 |  |
| R vOTC to R Cerebellum | 0.000 | 0 |  |
| R vOTC to R vOTC | -0.614 | 1 |  |
| L SMG to L vOTC (words) | 0.481 | 1 | 0.159 |
| L SMG to R Cerebellum (words) | 0.000 | 0 | -0.123 |
| L SMG to R vOTC (words) | 0.182 | 0.759 | 0.065 |
| L vOTC to L SMG (words) | 0.241 | 0.820 | 0.241 |
| L vOTC to R Cerebellum (words) | 0.262 | 0.937 | 0.314 |
| L vOTC to R vOTC (words) | 0.157 | 0.619 | 0.188 |
| R Cerebellum to L SMG (words) | 0.000 | 0 | 0.000 |
| R Cerebellum to L vOTC (words) | 0.000 | 0 | -0.118 |
| R Cerebellum to R vOTC (words) | -0.340 | 1 | -0.209 |
| R vOTC to L SMG (words) | -0.302 | 0.919 | -0.250 |
| R vOTC to L vOTC (words) | 0.000 | 0 | 0.000 |
| R vOTC to R Cerebellum (words) | 0.000 | 0 | 0.000 |
| L SMG to L vOTC (pseudowords) | 0.685 | 1 | 0.363 |
| L SMG to R Cerebellum (pseudowords) | 0.376 | 1 | 0.253 |
| L SMG to R vOTC (pseudowords) | 0.000 | 0 | -0.117 |
| L vOTC to L SMG (pseudowords) | 0.170 | 0.680 | 0.170 |
| L vOTC to R Cerebellum (pseudowords) | 0.000 | 0 | 0.052 |
| L vOTC to R vOTC (pseudowords) | 0.000 | 0 | 0.031 |
| R Cerebellum to L SMG (pseudowords) | 0.000 | 0 | 0.000 |
| R Cerebellum to L vOTC (pseudowords) | 0.000 | 0 | -0.118 |
| R Cerebellum to R vOTC (pseudowords) | 0.000 | 0 | 0.131 |
| R vOTC to L SMG (pseudowords) | 0.000 | 0 | 0.052 |
| R vOTC to L vOTC (pseudowords) | 0.000 | 0 | 0.000 |
| R vOTC to R Cerebellum (pseudowords) | 0.000 | 0 | 0.000 |
| L SMG (all trials) | 0.000 | 0 |  |
| L vOTC (all trials) | 0.260 | 1 |  |
| R Cerbellum (all trials) | 0.000 | 0 |  |
| R vOTC (all trials) | 0.114 | 0.839 |  |

**Supplementary Table S9**. DCM results for the extended reading network (left SMG, left vOTC, right vOTC, right cerebellum) for the effective vs. sham condition. Positive and negative values indicate the significant findings for the contrast of conditions: effective > sham (0 to 1; positive) and sham > effective (0 to -1; negative).

| **Connection** | **Parameter estimate** | **Posterior probability** |
| --- | --- | --- |
| L SMG to L SMG | 0.000 | 0 |
| L SMG to L vOTC | 0.000 | 0 |
| L SMG to R Cerebellum | 0.000 | 0 |
| L SMG to R vOTC | -0.058 | 1 |
| L vOTC to L SMG | 0.000 | 0 |
| L vOTC to L vOTC | 0.000 | 0 |
| L vOTC to R Cerebellum | 0.000 | 0 |
| L vOTC to R vOTC | 0.043 | 1 |
| R Cerebellum to L SMG | 0.048 | 1 |
| R Cerebellum to L vOTC | 0.000 | 0 |
| R Cerebellum to R Cerebellum | -0.028 | 0.559 |
| R Cerebellum to R vOTC | 0.082 | 1 |
| R vOTC to L SMG | 0.000 | 0 |
| R vOTC to L vOTC | 0.000 | 0 |
| R vOTC to R Cerebellum | 0.000 | 0 |
| R vOTC to R vOTC | -0.099 | 1 |
| L SMG to L vOTC (words) | 0.000 | 0 |
| L SMG to R Cerebellum (words) | 0.077 | 0.488 |
| L SMG to R vOTC (words) | 0.000 | 0 |
| L vOTC to L SMG (words) | 0.000 | 0 |
| L vOTC to R Cerebellum (words) | 0.000 | 0 |
| L vOTC to R vOTC (words) | 0.000 | 0 |
| R Cerebellum to L SMG (words) | 0.000 | 0 |
| R Cerebellum to L vOTC (words) | 0.000 | 0 |
| R Cerebellum to R vOTC (words) | -0.318 | 1 |
| R vOTC to L SMG (words) | 0.000 | 0 |
| R vOTC to L vOTC (words) | -0.207 | 1 |
| R vOTC to R Cerebellum (words) | 0.000 | 0 |
| L SMG to L vOTC (pseudowords) | 0.321 | 1 |
| L SMG to R Cerebellum (pseudowords) | 0.235 | 1 |
| L SMG to R vOTC (pseudowords) | 0.000 | 0 |
| L vOTC to L SMG (pseudowords) | 0.000 | 0 |
| L vOTC to R Cerebellum (pseudowords) | 0.000 | 0 |
| L vOTC to R vOTC (pseudowords) | 0.000 | 0 |
| R Cerebellum to L SMG (pseudowords) | -0.118 | 0.530 |
| R Cerebellum to L vOTC (pseudowords) | -0.404 | 1 |
| R Cerebellum to R vOTC (pseudowords) | -0.390 | 1 |
| R vOTC to L SMG (pseudowords) | 0.000 | 0 |
| R vOTC to L vOTC (pseudowords) | -0.168 | 0.710 |
| R vOTC to R Cerebellum (pseudowords) | -0.204 | 1 |
| L SMG (all trials) | 0.000 | 0 |
| L vOTC (all trials) | 0.000 | 0 |
| R Cerbellum (all trials) | 0.000 | 0 |
| R vOTC (all trials) | 0.000 | 0 |


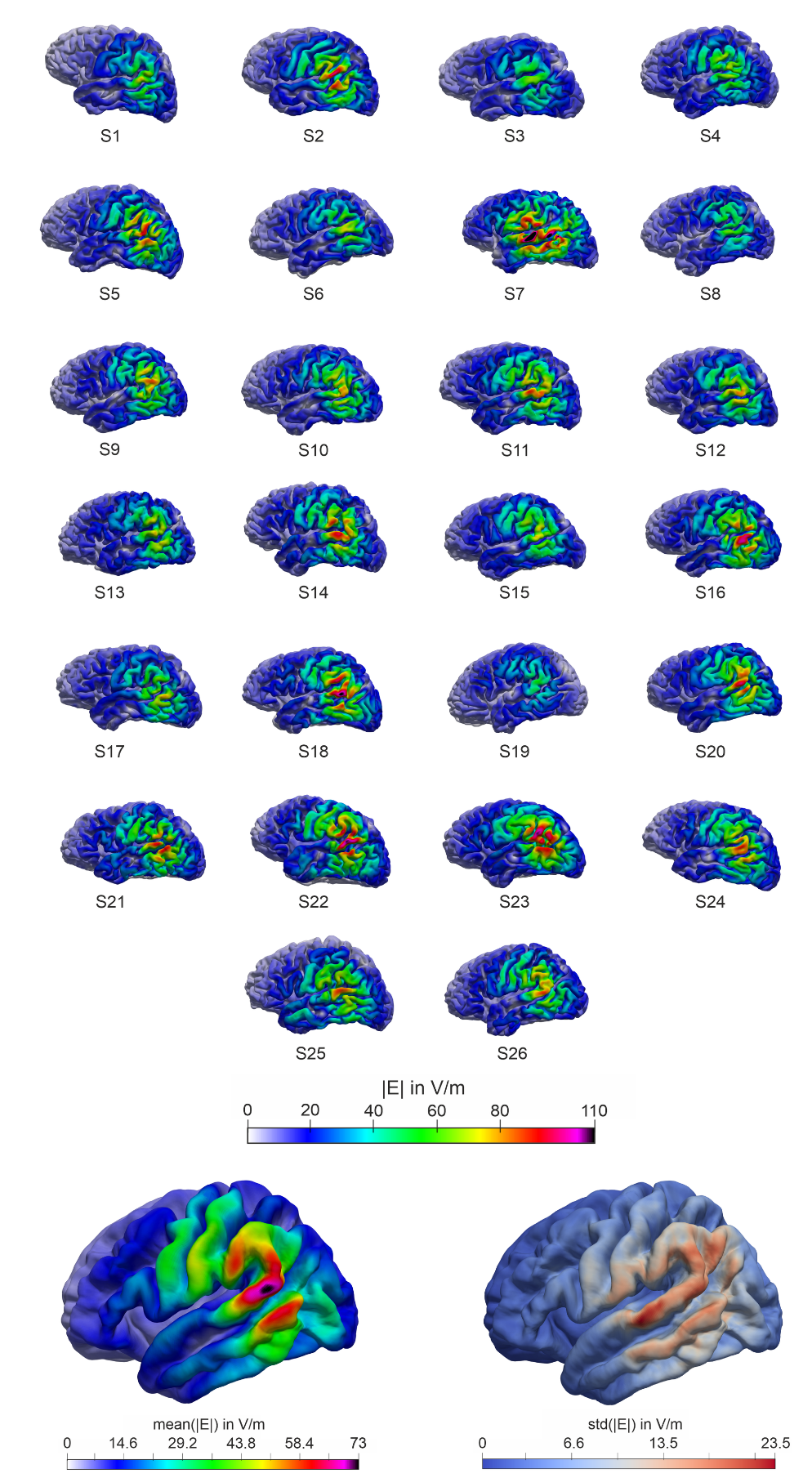


**Supplementary Figure S1.** Individual e-field simulation results and average e-field and standard deviation. Colours indicate the electrical field in V/m.
